# Glucose starvation causes ferroptosis-mediated lysosomal dysfunction

**DOI:** 10.1016/j.isci.2024.109735

**Published:** 2024-04-12

**Authors:** Kenji Miki, Mikako Yagi, Dongchon Kang, Yuya Kunisaki, Koji Yoshimoto, Takeshi Uchiumi

**Affiliations:** 1 Department of Clinical Chemistry and Laboratory Medicine, Graduate School of Medical Sciences, Kyushu University, Higashi-ku, Fukuoka 812-8582, Japan; 2 Department of Neurosurgery, Graduate School of Medical Sciences, Kyushu University, Higashi-ku, Fukuoka 812-8582, Japan; 3 Department of Health Sciences, Graduate School of Medical Sciences, Kyushu University, Higashi-ku, Fukuoka 812-8582, Japan; 4 Kashiigaoka Rehabilitation Hospital, Fukuoka 813-0002, Japan; 5 Department of Medical Laboratory Science, Faculty of Health Sciences, Junshin Gakuen University, Fukuoka 815-8510, Japan

**Keywords:** Cellular physiology, Cell biology, Functional aspects of cell biology

## Abstract

Lysosomes, the hub of metabolic signaling, are associated with various diseases and participate in autophagy by supplying nutrients to cells under nutrient starvation. However, their function and regulation under glucose starvation remain unclear and are studied herein. Under glucose starvation, lysosomal protein expression decreased, leading to the accumulation of damaged lysosomes. Subsequently, cell death occurred via ferroptosis and iron accumulation due to DMT1 degradation. GPX4, a key factor in ferroptosis inhibition located on the outer membrane of lysosomes, accumulated in lysosomes, especially under glucose starvation, to protect cells from ferroptosis. ALDOA, GAPDH, NAMPT, and PGK1 are also located on the outer membrane of lysosomes and participate in lysosomal function. These enzymes did not function effectively under glucose starvation, leading to lysosomal dysfunction and ferroptosis. These findings may facilitate the treatment of lysosomal-related diseases.

## Highlights

• Glucose starvation decreased lysosomal function
• This led to cell death via ferroptosis due to DMT1 degradation
• Lysosome function maintenance enzymes were located on outer lysosome membrane

## Graphical abstract

**Figure:**
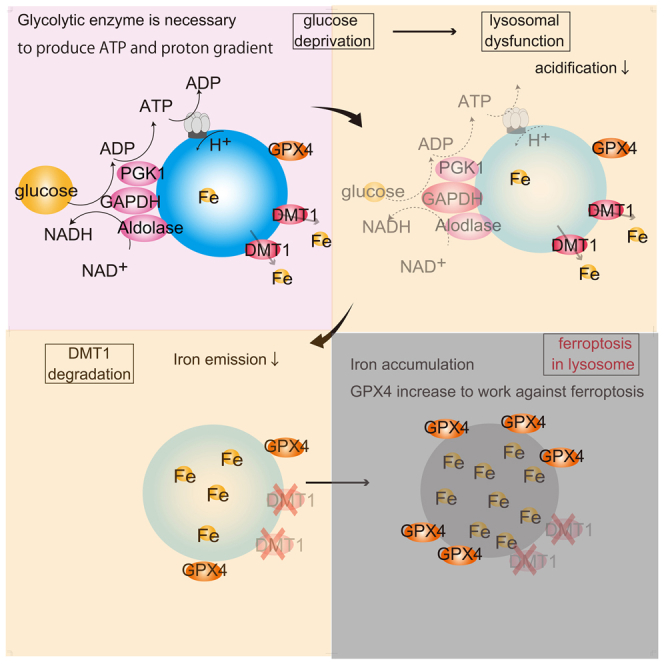

## Introduction

Nutrient deficiency, including glucose deficiency, is associated with various diseases. In some diseases, including cancer and Alzheimer’s disease, the micro-environment is glucose-starved.^1^^,^^2^ Under nutrient-deficient conditions, autophagy is enhanced to supply nutrients,^3^ and one of the key organelles in this process is the lysosome.^4^^,^^5^ Lysosomal dysfunction leads to Alzheimer’s disease.^6^ Lysosomes are the hub of metabolic signaling.^7^ Therefore, we postulated that the lysosome is a key organelle in glucose starvation. However, the function and regulation of lysosomes under glucose starvation remain unclear. Elucidation of the mechanisms by which lysosomes function under nutrient deficiency can facilitate the development of treatments for various diseases. Therefore, this study focused on elucidating the activity of lysosomes under glucose starvation.

Autophagy is enhanced under glucose-starved conditions.^8^ Additionally, alterations in the expression changes AMPK and ULK1 have been observed.^9^ Lee et al. reported that selective lysosomal membrane turnover is induced under glucose starvation.^10^ Considering these factors, we hypothesized that lysosomal function would increase under glucose starvation.

Ferroptosis is a mechanism of cell death involving iron accumulation.^11^^,^^12^ Two features of ferroptosis are lipid peroxidation and iron dependency.^12^^,^^13^^,^^14^^,^^15^ Poly-unsaturated fatty acids are (1) oxidized by reactive oxygen species arising from iron-dependent Fenton reactions,^15^ (2) sensitive to lipid peroxidation, and (3) essential for ferroptosis.^16^ Lysosomes play an important role in cellular iron homeostasis and contain DMT1, which excretes iron from lysosomes.^17^ Oxidised glutathione (GSSG) increases during ferroptosis,^18^ establishing its potential as a biomarker for ferroptosis. Moreover, GPX4, which inhibits membrane phospholipid hydroperoxide formation and ferroptosis,^19^^,^^20^^,^^21^ is present in the cytosol and nucleus,^22^ and decreases during ferroptosis. Herein, we aimed to determine how GPX4 functions and where it is expressed during ferroptosis.

mTOR is a lysosomal protein^23^^,^^24^ that regulates V-ATPase.^25^ Lysosomal acidification is regulated by V-ATPase, which produces ATP and enables protons to enter the lysosomal lumen in an ATP-dependent manner.^26^^,^^27^ mTOR hyperactivity impairs lysosomal function.^28^ Additionally, mTOR expression increases under adequate nutrient supply.^29^ Under glucose starvation, AMPK increases, decreasing mTOR expression.^29^

ATP-producing enzymes, including GAPDH, are present in the outer membrane of lysosomes, and are essential for lysosomal function.^30^ Therefore, we suspect that numerous enzymes, which play important roles in sensing and maintaining lysosomal function, are present in the outer membrane of lysosomes.

Herein, we examined the behavior of autophagy- and lysosomal-related proteins in cells under glucose starvation. Specifically, under glucose starvation, lysosomal function decreases, including cathepsin activity and lysosomal acidification. Following lysosomal dysfunction, iron accumulation and ferroptosis occur through DMT1 degradation, especially in lysosomes. GPX4 then accumulates on the outer membrane of lysosomes, protecting them from ferroptosis. The main reason lysosomal dysfunction occurs, in addition to mTOR accumulation, is because glycolytic enzymes in the outer membrane of lysosomes do not work efficiently under glucose starvation.

## Results

### Glucose starvation decreased lysosomal proteins

**Figure 1:**
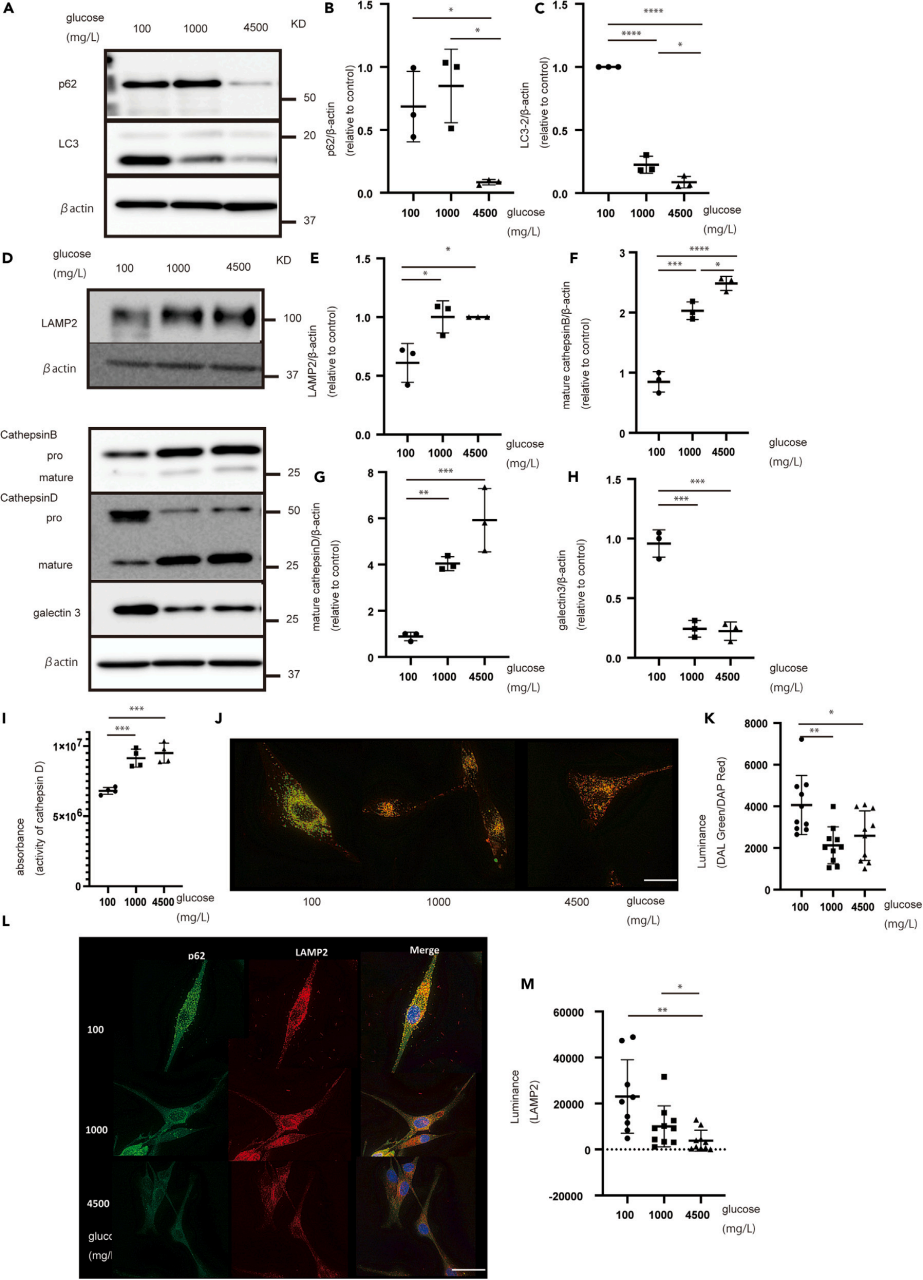
Glucose starvation increases the expression of autophagy-related proteins and decreases the expression of lysosomal proteins in U87 cells (A) Expression of autophagy-related proteins (p62 and LC3) under each glucose concentration for 72 h. (B and C) Quantification of (B) p62 and (C) LC3. (D) Expression of lysosomal proteins (mature cathepsin B and D, LAMP2, and LGALS3). (E–H) Quantification of (E) LAMP2, (F) mature cathepsin B, (G) mature cathepsin D, and (H) LGALS3. (I) Cathepsin D activity decreased under glucose starvation in U87 cells. (J and K) DALGreen/DAPRed fluorescence (J) showed lower intensity under glucose starvation and (K) its quantification in U87 cells. (L) Morphology of p62 and LAMP2 under glucose starvation. (M) Quantification of LAMP2 area in U87 cells. Scale bars: 20 μm. Values are presented as mean ± SD. One-way ANOVA and Turkey’s multiple comparison test were performed to assess 100 vs. 1,000 vs. 4,500 mg/L of glucose. ^∗^*p* < 0.05; ^∗∗^*p* < 0.01; ^∗∗∗^*p* < 0.001; ^∗∗∗∗^*p* < 0.0001.

We postulated that glucose starvation increases autophagy and lysosomal activity. After 72 h of culture under glucose starvation, the levels of autophagy-related proteins (p62 and LC3) accumulated, thereby suggesting lysosomal dysfunction (Figures 1A–1C, S1A–S1C, and S1F–S1H). The levels of lysosomal proteins (mature cathepsin B and D and LAMP2) decreased. Moreover, LGALS3, a marker of lysosomal damage, increased under glucose starvation (Figures 1D‒1H, S1A, S1D‒S1F, and S1I‒S1K). To confirm the increased and decreased expression of autophagy- and lysosomal-related proteins, respectively, we examined lysosomal function under glucose starvation.

### Lysosomal activity decreased under glucose starvation and led to abnormal lysosomal accumulation

To determine lysosomal activity, we measured cathepsin D activity. Cathepsin D activity decreased when cells were subjected to glucose starvation (Figure 1I). Additionally, DQ-BSA (lysosomal protease activity) and DALGreen/DAPRed (lysosomal acidification) were used, as previously described.^30^ DQ-BSA accumulated in lysosomes via endocytosis and was hydrolyzed by lysosomal protease into small peptides, which do not quench fluorescence.^31^ Lysosomal protease activity can be assessed by measuring the rate of increase in fluorescence, as previously described.^30^ DALGreen fluorescence (auto-lysosomal vesicles) is enhanced at an acidic pH and is suitable for monitoring the autophagy degradation stage, namely the auto-lysosome stage. Conversely, DAPRed (auto-phagosomes) has a pH-independent intensity throughout the autophagy process.^30^ DQ-BSA absorbance decreased under glucose starvation (Figure S1L). Lysosomal acidification (pH level) and degradation were checked using DALGreen/DAPRed. Figures 1J and 1K shows the accumulation of both auto-phagosomes and auto-lysosomes under glucose starvation, implying that autophagic degradation is inhibited by lysosomal dysfunction.

To examine lysosomal acidification, we used two fluorescently tagged probes: the pH-sensitive tetramethylrhodamine-dextran and Oregon Green 488, with a pKa of 4.7, both of which suitable for measuring the acidic pH of the lysosomal lumen. The emission was separately determined in individual lysosomes, and the fluorescence ratio was measured, enabling changes in lysosomal pH to be monitored (more acidic shows lower green/red ratio, whereas less acidic shows higher green/red).^30^^,^^32^ In the glucose-starved condition, both cell lines, MEF and U87, showed reduced lysosomal acidification (Figure S1M–S1P). Barral et al. focused on the relationship between lysosomal morphology and lysosomal function and reported that lysosomal morphology affects lysosomal function.^33^ We examined lysosomal morphology. Under glucose starvation, p62 and LAMP2 staining increased, implying a change in lysosomal morphology. Some p62 and LAMP2 staining merged (Figures 1L and 1M). Moreover, the stained areas of GALS3 and LC3 were larger under glucose starvation than those under other conditions (Figures S2A‒S2C). These results suggest lysosomal dysfunction under glucose starvation.

### Glucose starvation increased the expression of autophagy-related proteins via the AKT/AMPK/ULK1 pathway

We examined the expression of proteins up-stream of p62 and LC3. Under glucose starvation, autophagy is enhanced, and the expression of AMPK and ULK1, which are up-stream of p62, is altered.^8^^,^^9^ Under glucose starvation, *p*-AKT (Ser473/Th308) decreased; however, the expression of AKT did not change (Figures S2D‒S2G). Moreover, *p*-AMPK and AMPK increased under glucose starvation. The results for *p*-ULK1 and ULK1 were the same as those for *p*-AMPK and AMPK in that *p*-ULK1 and ULK1 also increased under glucose starvation (Figures S2H‒S2K). The first reaction of cells under glucose starvation was decreased *p*-AKT expression, followed by increased AMPK, ULK1, and p62 expression (Figure S2L), consistent with previous reports.^8^^,^^9^

### Glucose starvation altered iron homeostasis and induced ferroptosis

**Figure 2:**
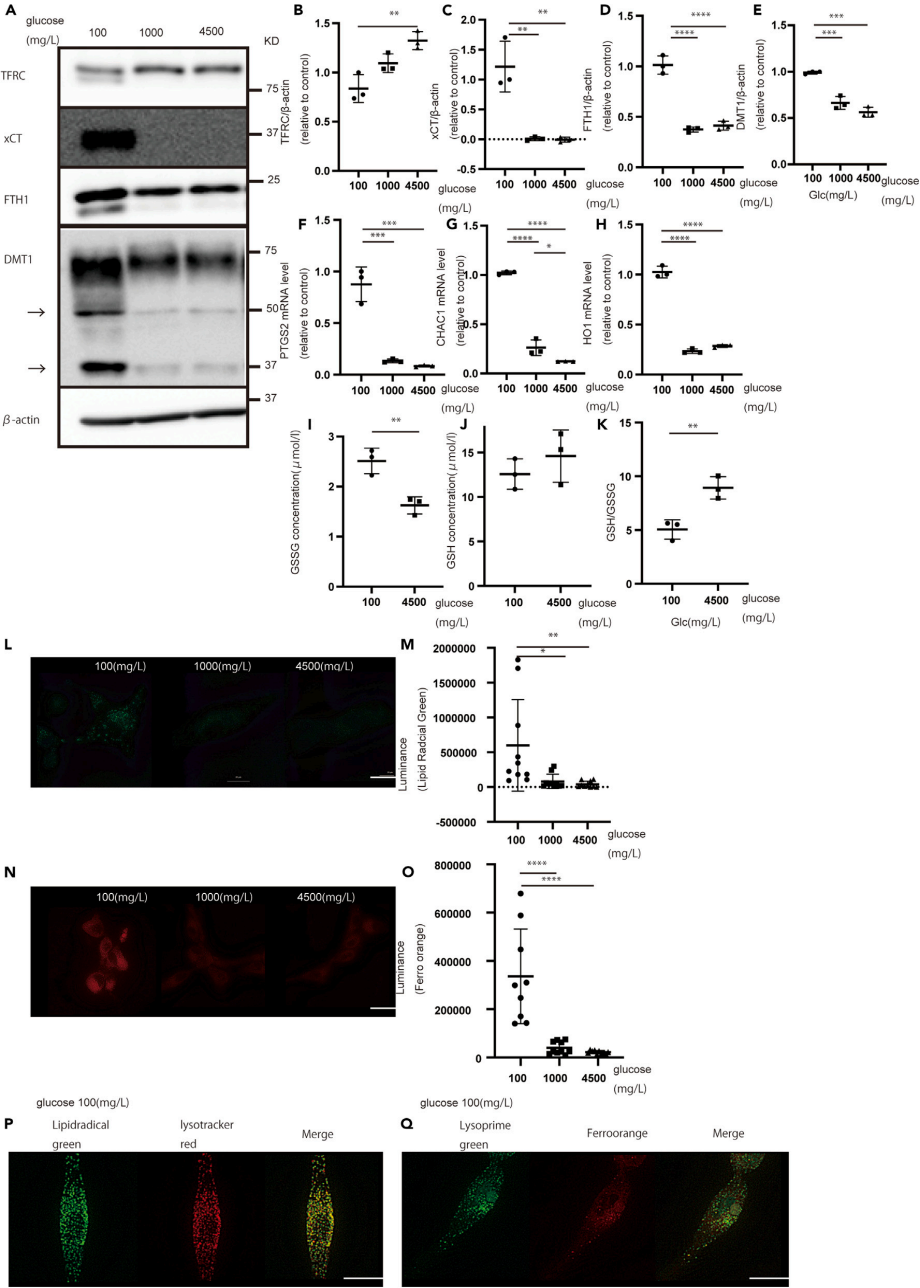
Glucose starvation induces significant changes in iron-related proteins (A) Expression of iron-related proteins in U87 cells under each glucose concentration for 72 h. (B–E) Quantification of (B) TFRC, (C) XCT, (D) FTH1, and (E) DMT1. (F–H) Quantification of (F) PTGS2, (G) CHAC1, and (H) HO-1. (I–K) Quantification of (I) GSSG, (J) GSH, and (K) GSH/GSSG. (L) LipiRADICAL Green fluorescence intensity was higher under glucose starvation. (M) Quantification of LipiRADICAL Green fluorescence. (N) FerroOrange fluorescence under glucose starvation and its quantification. (O and P) LipiRADICAL Green and LysoTracker red co-localization. (Q) LysoPrime Green and FerroOrange co-localization. Scale bars: 20 μm. Values are presented as mean ± SD. One-way ANOVA and Tukey’s multiple comparison test were performed to assess 100 vs. 1,000 vs. 4,500 mg/L of glucose. ^∗^*p* < 0.05; ^∗∗^*p* < 0.01; ∗∗∗*p* < 0.001; ^∗∗∗∗^*p* < 0.0001.

We examined the effect of decreased lysosomal protein expression under glucose starvation. There is a relationship between lysosomes and iron dynamics.^34^ The levels of iron-related proteins (DMT1, FTH1, TFRC, and XCT) were considerably altered. TFRC expression decreased, whereas DMT1, FTH1, and XCT expression increased (Figures 2A‒2E). Moreover, the DMT1 band changed under glucose starvation, implying DMT1 dysfunction (Figure 2A). Considering these factors, iron homeostasis changes, suggesting that iron accumulates under glucose starvation. After a 72-h incubation under glucose-starved conditions, cells exhibited a weakened state and detached, thereby implying cell death (Figures S3A and S3B). Therefore, we hypothesized that glucose starvation induces ferroptosis and checked the mRNA levels. As expected, ferroptosis markers,^12^ including CHAC1, HO-1, and PTGS2, were markedly increased under glucose starvation (Figures 2F‒2H and S3C‒S3G). As GSSG is related to ferroptosis,^18^ we also examined oxidative glutathione levels. Under glucose starvation, the GSSG ratio increased (Figures 2I‒2K).

LipiRADICAL Green and FerroOrange were used to investigate the occurrence of ferroptosis. LipiRADICAL Green indicates the accumulation of lipid radicals, implying ferroptosis, whereas FerroOrange indicates iron accumulation.^35^ Under glucose starvation, lipid radicals and iron accumulated (Figures 2L‒2O and S3H‒S3J). As a positive control, we checked the intensity of LipiRADICAL Green and FerroOrange using RSL3 (a ferroptosis inducer) (Figures S3K‒S3N). Moreover, we confirmed the decrease in lipid radical accumulation using deferoxamine (an iron chelator and ferroptosis inhibitor)^11^^,^^36^ (Figures S3O and S3P). Moreover, the ferroptosis inhibitor Fer1 inhibited cell death under glucose-starved conditions (Figures S3Q–S3V). The localization of ferroptosis overlapped with that of lysosomes. LipiRADICAL Green and LysoTracker Red staining were merged, in addition to LysoPrime Green (lysosomes) and FerroOrange staining (Figures 2P and 2Q). These results suggest that ferroptosis occurs in lysosomes under glucose starvation.

### Ferroptosis occurred after lysosomal dysfunction

**Figure 3:**
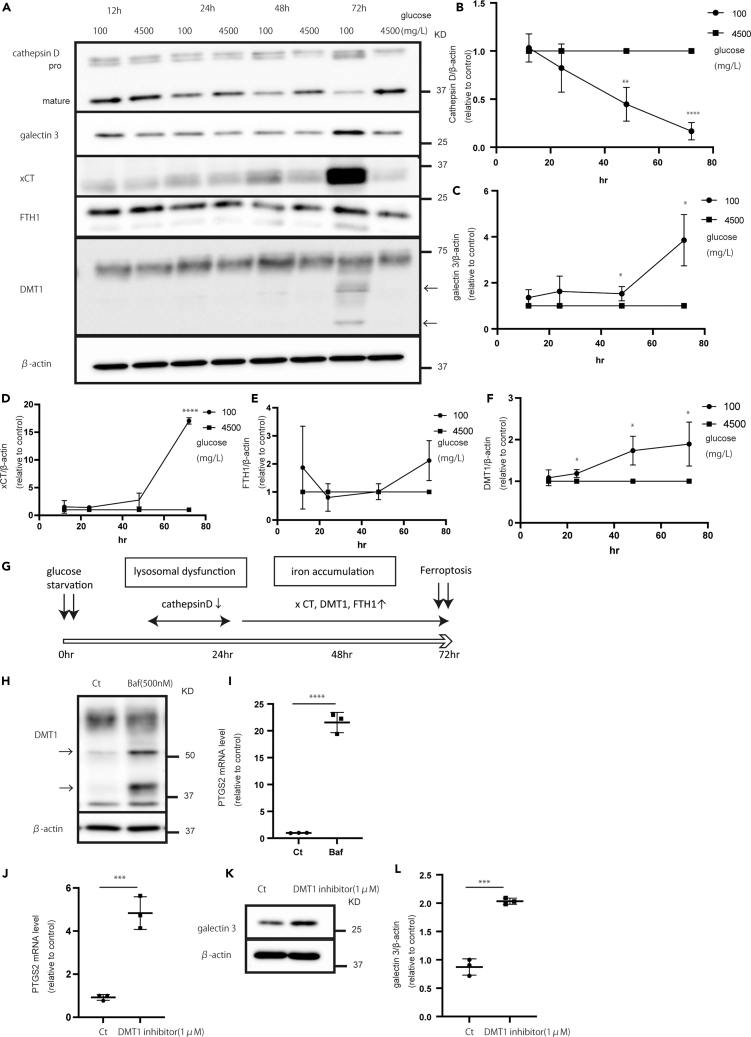
Time-course experiments of lysosomal function and iron dynamics (A) Expression of lysosomal- and iron-related proteins in U87 cells. (B–F) Quantification of (B) mature cathepsin D, (C) LGALS3, (D) XCT, (E) FTH1, and (F) DMT1. (G) Time-course experiments of lysosomal dysfunction and iron dynamics. Values are presented as mean ± SD. One-way ANOVA and Tukey’s multiple comparison test were performed to assess 100 vs. 4,500 mg/L of glucose. ^∗^*p* < 0.05; ^∗∗^*p* < 0.01; ^∗∗∗∗^*p* < 0.0001. (H and I) Expression of (H) DMT1 after the addition of BafA1 (500 nM) for 48 h and (I) quantification of PTGS2. (J) Quantification of PTGS2 using DMT1 blocker (1 μM) for 72 h. (K and L) Expression of (K) LGALS3 and (L) its quantification using DMT1 blocker. Values are presented as mean ± SD. One-way ANOVA and Tukey’s multiple comparison test were performed to assess 100 vs. 4,500 mg/L of glucose. ^∗^*p* < 0.05; ^∗∗^*p* < 0.01; ^∗∗∗∗^*p* < 0.0001.

We hypothesized that lysosomal dysfunction induces ferroptosis. To determine whether lysosomal dysfunction occurs before iron accumulation, we conducted a time-course experiment. The time-course experiment showed that from 1224 h, cathepsin D expression decreased. After 72 h, LGALS3 expression increased, suggesting that lysosomal damage increased (Figures 3A‒3C). However, the expression of ferroptosis-related proteins (DMT1, FTH1, and XCT) increased after 72 h (Figures 3A and 3D‒3F). Considering these results, lysosomal dysfunction occurred earlier, iron homeostasis was altered, and ferroptosis occurred (Figure 3G).

To confirm that lysosomal dysfunction induced ferroptosis, we used BafA1 (a V-ATPase inhibitor). Ferroptosis-related mRNA levels, including CHAC1 and PTGS2, were especially elevated (Figures 3I, S4A, and S4B). These results suggest that lysosomal dysfunction induces ferroptosis. Moreover, we elucidated the mechanism by which lysosomal dysfunction induced ferroptosis. Under glucose starvation, the DMT1 band in Figures 2A and 3A changed: namely, the band became broad, and a fragmented band was observed. DMT1 excretes iron from lysosomes into the cytoplasm, and DMT1 degradation induces iron accumulation in lysosomes. This suggests that lysosomal dysfunction induces DMT1 degradation. BafA1 induces DMT1 fragmentation (Figure 3H). Moreover, ferroptosis-related mRNA levels and LGALS3 expression were increased upon DMT1 inhibitor treatment (Figures 3J‒3L and S4C). Therefore, DMT1 dysfunction causes ferroptosis and lysosomal dysfunction. These results suggest that lysosomal dysfunction induces DMT1 fragmentation, leading to iron accumulation in lysosomes.

### GPX4 increased and was present in lysosomes under glucose starvation

**Figure 4:**
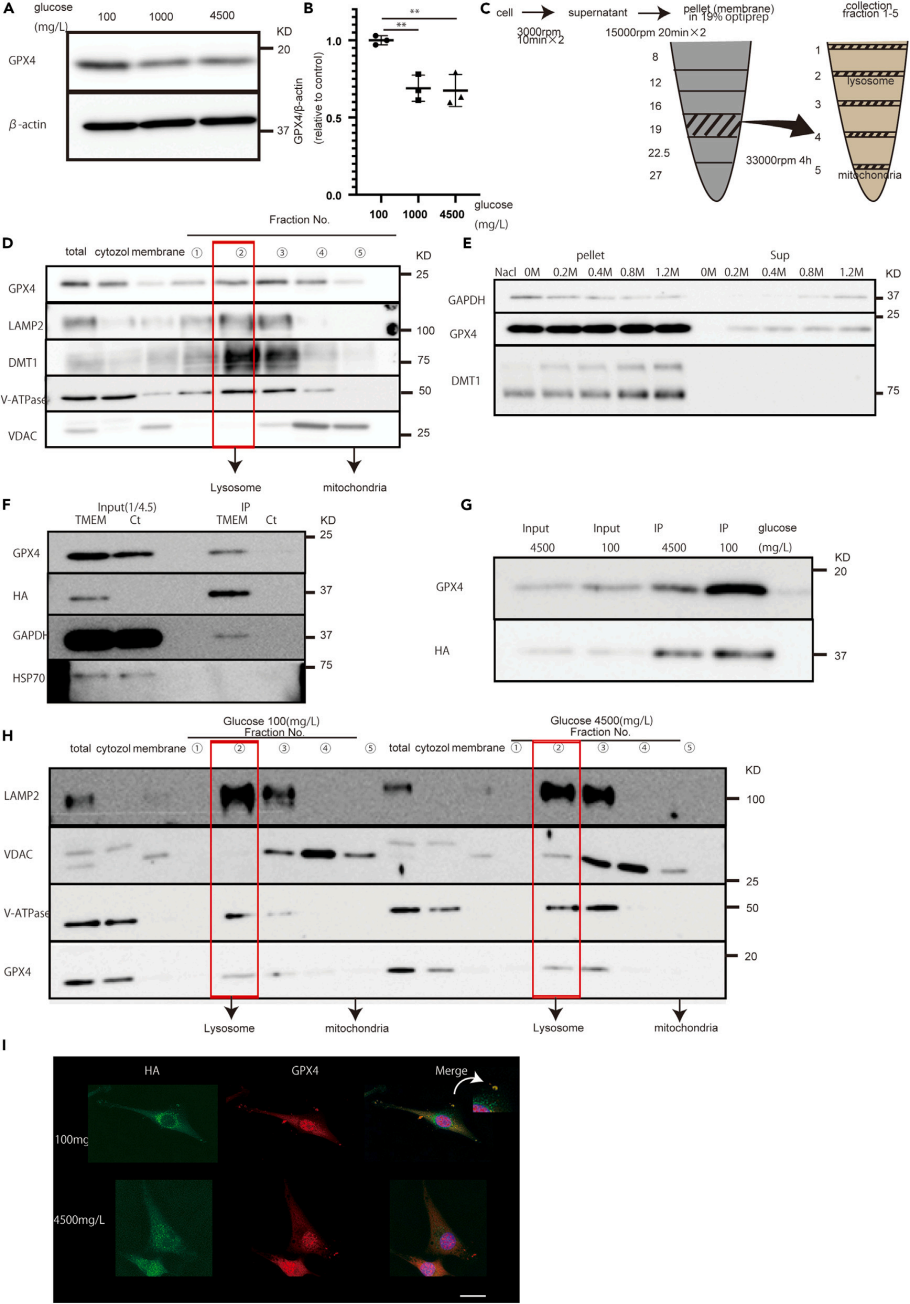
Increased GPX4 expression and localization in the lysosomal fraction of U87 cells under glucose starvation (A) Expression of (A) GPX4 and (B) its quantification under each glucose concentration for 72 h. (C) Schematic of the purification of lysosomes by centrifugation. (D) Protein localization using fractionation experiments. (E) Protein expression at different salt concentrations. (F) Immunoprecipitation using TMEM192-transfected cells and lysosomal expression. (G) Immunoprecipitation using TMEM192-transfected cells under different glucose concentrations for 72 h. (H) Fraction difference between glucose starvation and glucose-rich conditions. (I) GPX4 immunostaining shows only glucose starvation, and co-localization of GPX4 and HA. Scale bars: 20 μm. Values are presented as mean ± SD. One-way ANOVA and Tukey’s multiple comparison test were performed to assess 100 vs. 1,000 vs. 4,500 mg/L of glucose. ^∗∗^*p* < 0.01.

GPX4 inhibits ferroptosis,^21^ and its expression decreases during ferroptosis.^37^ GPX4 expression increased under glucose starvation (Figures 4A, 4B, S4D, and S4E). We hypothesized that GPX4 is present in lysosomes and protects cells from ferroptosis. Therefore, we conducted fractionation experiments to elucidate the location of lysosomes. Fractionation experiments have confirmed a compartment between lysosomes and mitochondria.^30^^,^^38^

In this study, fractionation experiments showed that LAMP2 and V-ATPase were present in the lysosomal fraction, whereas voltage-dependent anion-selective channels were present in the mitochondrial fraction (Figure 4C,D). Moreover, DMT1 and GPX4 were detected in the lysosomal fraction (Figure 4D). To confirm the presence of GPX4 in lysosomes, we checked the expression of proteins detached at different salt concentrations. GAPDH is present in the outer membrane of lysosomes and detaches according to the salt concentration. GPX4 had similar results to GAPDH,^30^ suggesting that GPX4 moderately adheres to the outer membrane of lysosomes, while DMT1 strongly adheres to the outer membrane of lysosomes (Figure 4E). Moreover, we conducted immunoprecipitation experiments using TMEM192-transfected cells. GAPDH and GPX4 were found in the lysosomal fraction, whereas HSP70 was not (Figure 4F).

### Increased GPX4 accumulation in lysosomes under glucose starvation

We previously demonstrated that GPX4 is present in the outer membrane of lysosomes and checked whether GPX4 accumulates under glucose starvation. Immunoprecipitation revealed a 2.3-times increase in GPX4 accumulation in lysosomes under glucose starvation (Figure 4G). Moreover, under glucose starvation, GPX4 was enriched in the lysosomal fraction (Figure 4H). Finally, immunostaining showed that under glucose starvation, GPX4 and lysosomes merged; however, there was no merging under glucose-rich conditions (Figure 4I). These results suggest that GPX4 accumulates in the outer membrane of lysosomes under glucose starvation.

### GPX4 inhibition causes cell death under glucose starvation

**Figure 5:**
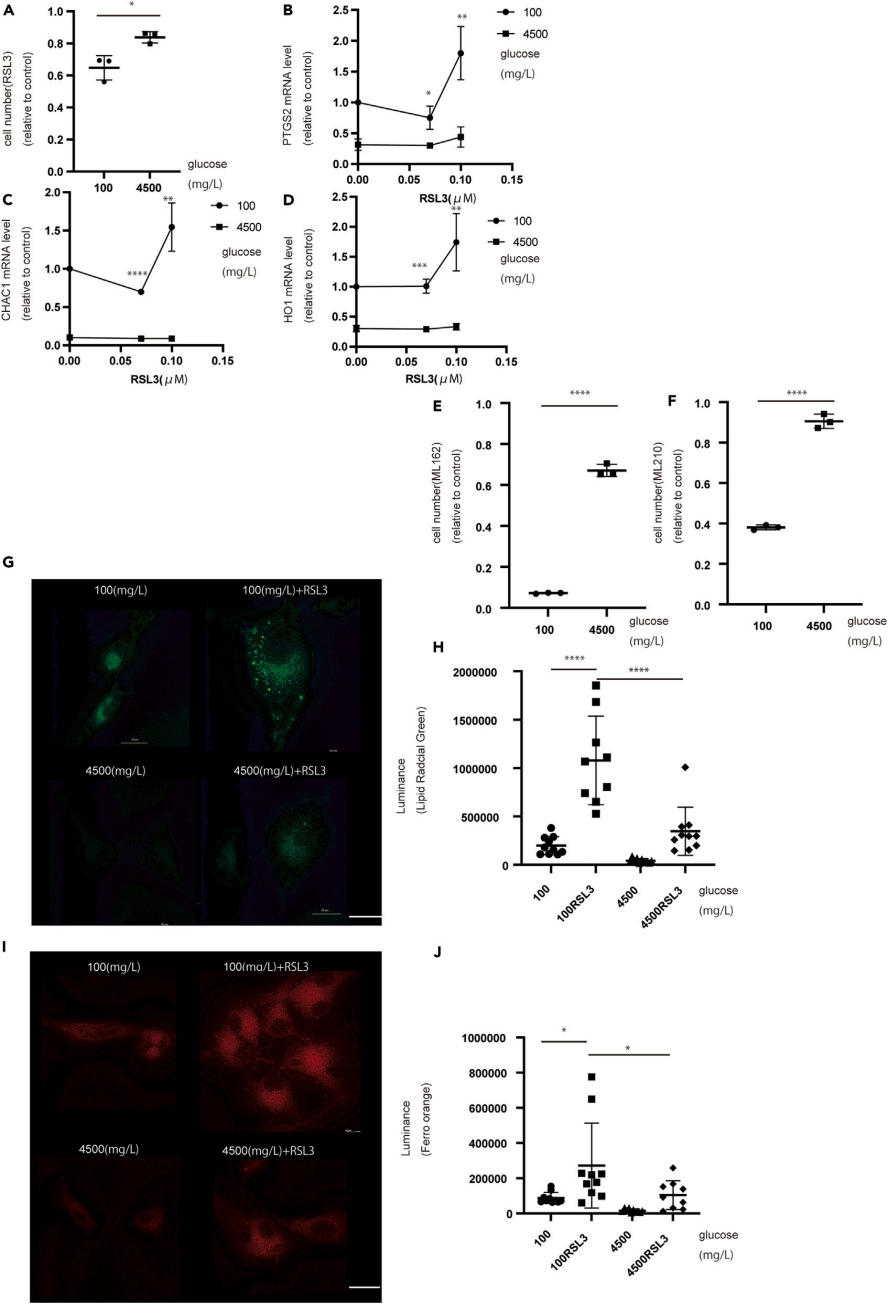
GPX4 inhibitors promote cell death under glucose starvation (A) Cell number at each concentration of glucose (72 h) and RSL3 (24 h) in U87 cells. (B–D) Quantification of (B) PTGS2, (C) CHAC1, and (D) HO-1 at each concentration of glucose (72 h) and RSL3 (48 h). (E and F) Cell number at each concentration of glucose (72 h) and (E) ML162 and (F) ML210. (G) LipiRADICAL Green staining increased with RSL3, especially under glucose starvation. (H) Quantification of LipiRADICAL Green staining after 72 h. (I) FerroOrange staining increased with RSL3, especially under glucose starvation. (J) Quantification of FerroOrange staining. Scale bars: 20 μm. Values are presented as mean ± SD. One-way ANOVA and Tukey’s multiple comparison test were performed to assess 100 vs. 4,500 mg/L of glucose + RSL3. ^∗^*p* < 0.05; ^∗∗^*p* < 0.01; ^∗∗∗^*p* < 0.001; ^∗∗∗∗^*p* < 0.0001.

Considering that GPX4 may protect cells from ferroptosis, we confirmed this phenomenon using GPX4 inhibitors (ML162, ML210, and RSL3). As expected, RSL3 induced greater cell death under glucose starvation than under normal conditions (Figure 5A). Ferroptosis-related mRNA levels, including CHAC1, HO-1, and PTGS2, also increased under glucose starvation (Figures 5B‒5D). ML162 and ML210 (also GPX4 inhibitors) induced cell death, especially under glucose starvation (Figures 5E and 5F). LipiRADICAL Green and FerroOrange showed greater accumulation under glucose starvation than under normal conditions (Figures 5G‒5J). Under glucose starvation, when using RSL3, the location of LipiRADICAL Green accumulation was similar to that of LysoTracker Red (lysosomes) (Figure S4F). Moreover, LysoPrime Green (lysosomes) and FerroOrange were merged; therefore, ferroptosis occurred in lysosomes (Figure S4G). In ML210, lipid radical accumulation showed that ferroptosis occurred under glucose starvation (Figures S4H and S4I). These results suggest that GPX4 is essential for lysosomal function, especially under glucose starvation, and that its inhibition causes ferroptosis.

### mTOR increased under glucose starvation and caused lysosomal damage

**Figure 6:**
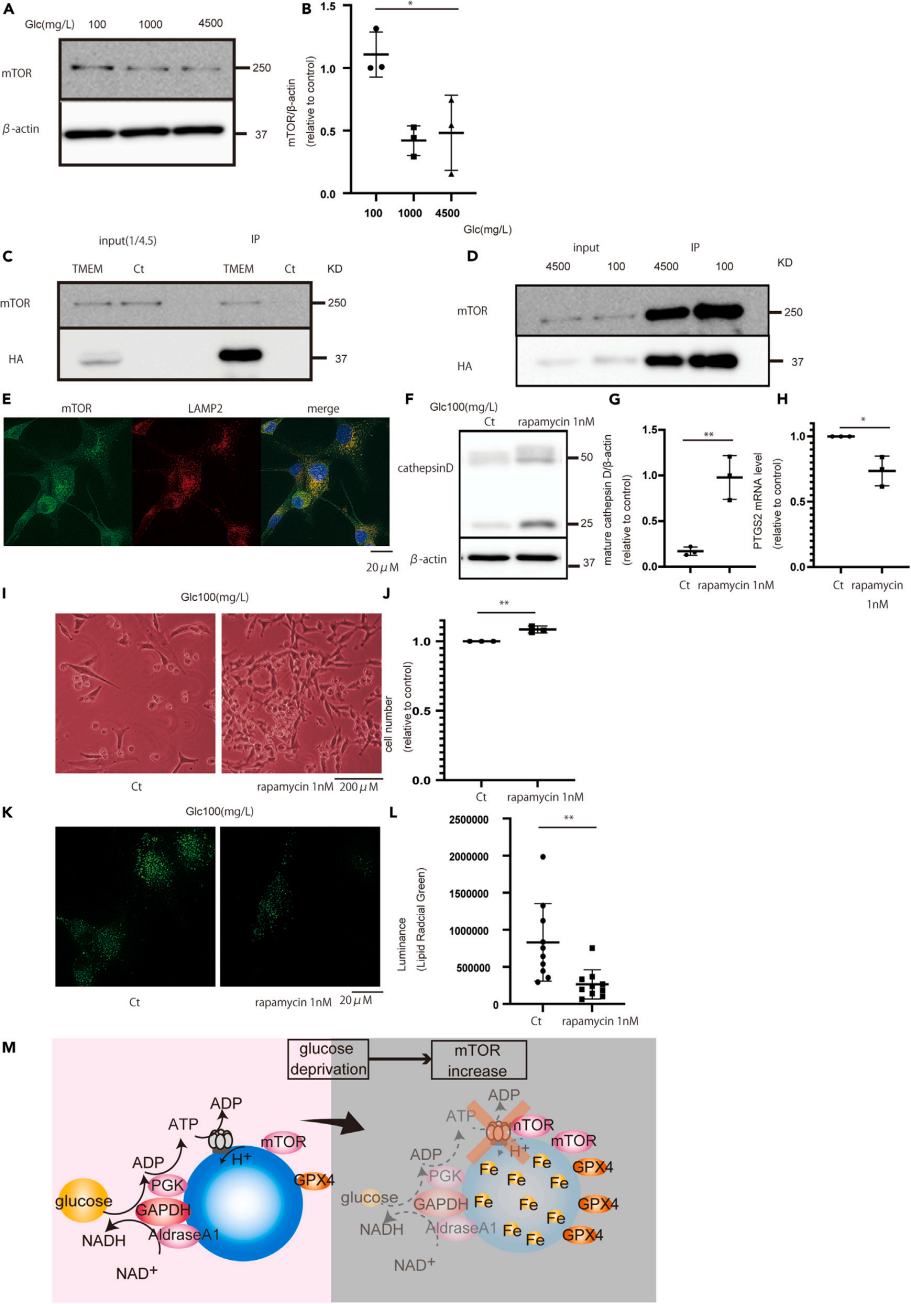
mTOR increases under glucose starvation causing lysosomal dysfunction (A) Expression of mTOR: mTOR increases under glucose starvation for 72 h in U87 cells. (B) Quantification of mTOR. (C) Immunoprecipitation of mTOR only in TMEM192-transfected cells. (D) Lysosomal mTOR increased under glucose starvation for 72 h. (E) mTOR and LAMP2 co-localization. (F) Rapamycin increased the expression of mature cathepsin D. (G) Quantification of cathepsin D expression after 72 h. (H) PTGS2 decreased under glucose starvation. (I and J) Cell number increased with (J) rapamycin treatment and (K) its quantification. (K and L) LipiRADICAL Green staining decreased with (L) rapamycin treatment and (M) its quantification after 72 h. (M) Schematic of changes in mTOR under glucose starvation. Scale bars: 20 μm. Values are presented as mean ± SD. One-way ANOVA and Tukey’s multiple comparison test were performed to assess 100 vs. 1,000 vs. 4,500 mg/L of glucose or control vs. rapamycin treatment. ^∗^*p* < 0.05; ^∗∗^*p* < 0.01.

An important enzyme related to lysosomal function is mTOR, which regulates V-ATPase.^25^ mTOR is decreased under glucose starvation.^29^ However, mTOR expression increased under glucose starved condition (Figures 6A and 6B). mTOR is present in lysosomes, and under glucose starvation, lysosomal mTOR increased more than that under normal conditions (Figures 6C‒6E). This contradiction may cause lysosomal damage, because mTOR hyperactivity impairs lysosomal function.^28^ Therefore, mTOR inhibitors, such as rapamycin, may improve lysosomal function under glucose starvation. As expected, rapamycin increased mature cathepsin D expression under glucose starvation and decreased PTGS2 mRNA levels (Figures 6F‒6H). Moreover, although the effect was minimal, rapamycin inhibited cell death under glucose starvation (Figures 6I and 6J). There was low accumulation of lipid radicals when rapamycin was used (Figures 6K and 6L). The influence of mTOR under glucose starvation is shown in Figure 6M.

### Glycolytic enzymes are present in lysosomes, and glucose is essential for lysosomal function

**Figure 7:**
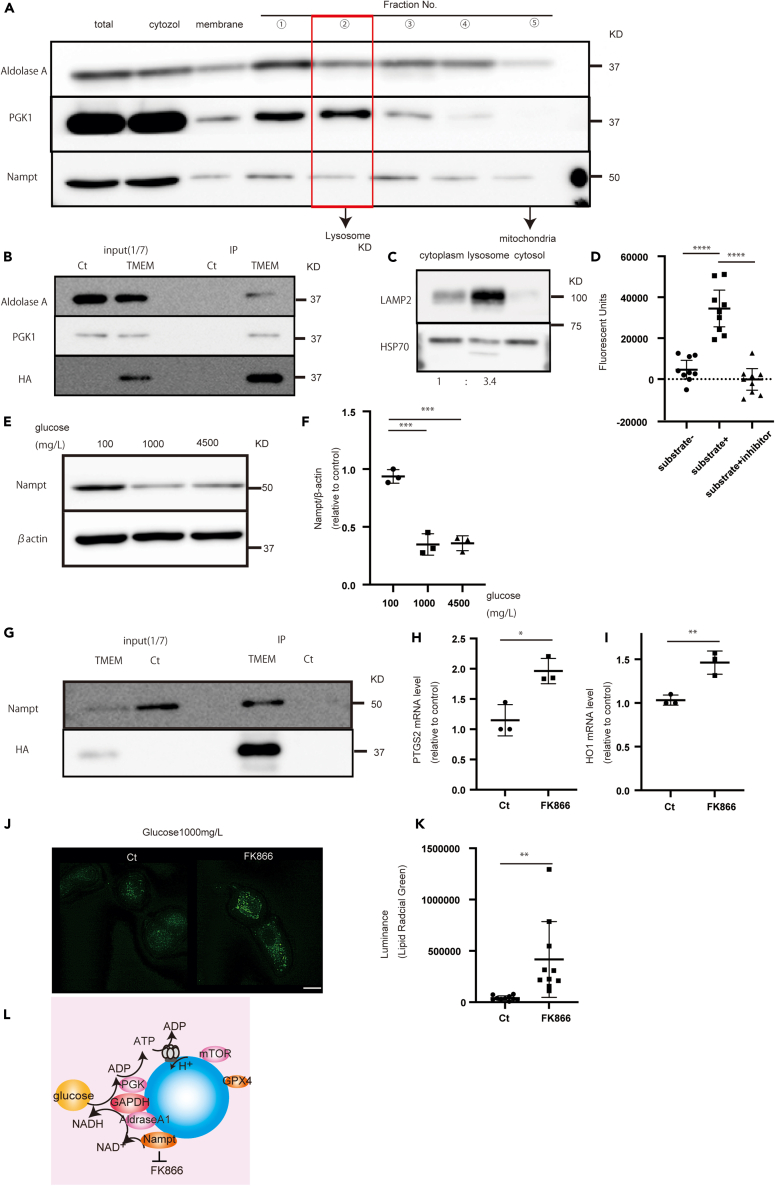
ALDOA, PGK1, and NAMPT are present in lysosomes and are important for lysosomal function and FK866 induces ferroptosis (A) ALDOA, PGK1, and NAMPT are present in the lysosomal fraction of U87 cells. (B) Immunoprecipitation experiments showed that ALDOA and PGK1 are present in TMEM192-transfected cells. (C) LAMP2 and HSP70 expression in each fraction of purified lysosomes. (D) Fluorescence in each group with or without substrate and inhibitor. (E) NAMPT increased under glucose starvation for 72 h. (F) NAMPT quantification. (G) NAMPT was present in TMEM192-transfected cells. (H and I) Expression of (H) PTGS2 and (I) HO-1 after the addition of FK866 (48 h). (J) LipiRADICAL Green staining decreased with increasing FK866. (K) Quantification of LipiRADICAL Green fluorescence. (L) Schematic of NAMPT localization in lysosomes. Scale bars: 20 μm. Values are presented as mean ± SD. One-way ANOVA and Tukey’s multiple comparison test were performed to assess 100 vs. 1,000 vs. 4,500 mg/L of glucose or control vs. FK866 treatment. ^∗^*p* < 0.05; ^∗∗^*p* < 0.01; ^∗∗∗^*p* < 0.001.

We investigated the mechanism by which glucose deprivation worsens lysosomal function. ATP-producing enzymes, including GAPDH and PGK1, are present in the lysosomal membrane (Figures 4C, 4D, 7A, and 7B). Therefore, we hypothesized that other glycolytic kinases are present in lysosomes, and that the lysosomal membrane is important, because many enzymes, including glycolytic enzymes, maintain lysosomal function. One glycolytic enzyme, ALDOA, was also present in the lysosomal membrane (Figures 7A and 7B).

These results suggest that glucose is important for maintaining lysosomal function, because numerous glycolytic enzymes are present in the lysosomal membrane. To test this hypothesis, we extracted lysosomes without mitochondria and added F1,6BP, a substrate for ALDOA, to confirm the presence of ALDOA/GAPDH/PGK1 around lysosomes. F1,6BP treatment increased ATP production, whereas treatment without F1,6BP, or with a GAPDH inhibitor, decreased ATP production (Figures 7C and 7D). Moreover, PGK1 decreased under glucose starvation, which may affect ATP production (Figures S5A and S5B).

### NAMPT is also present in lysosomes and is important for lysosomal function

NAMPT, an enzyme of the NAD pathway, is important for lysosomal function.^30^ Under glucose starvation, NAMPT expression was altered (Figures 7E and 7F). NAMPT was present in the lysosomal membrane (Figure 7G). FK866 (an NAMPT inhibitor) increased the mRNA levels of HO-1 and PTGS2 (Figures 7H and 7I). FK866 increased LipiRADICAL Green staining, indicating the occurrence of ferroptosis (Figures 7J and 7K). These results suggest that NAMPT is important for lysosomal function (Figure 7L).

### Lysosomal function did not decrease under glutamine deprivation

To confirm the importance of glucose in lysosomal function, we examined changes under glutamine deprivation. Notably, under glutamine deprivation, mature cathepsin B and D expression increased (Figures S5C‒S5E). DAPRed/DALGreen staining showed that lysosomal function did not change (Figures S5F and S5G). Therefore, lysosomal dysfunction was caused only by glucose starvation or FK866 injection, confirming the importance of glucose in lysosomal function.

## Discussion

Lysosomes are the hub of metabolic signaling.^7^ In this study, we report the mechanism of lysosomal function, especially under glucose starvation. To our knowledge, there are no reports on the long-term changes in lysosomal function under glucose starvation in which cells can survive, similar to the micro-environments in the human body. According to our findings, under glucose starvation: (1) Lysosomal dysfunction occurs; (2) ferroptosis occurs after lysosomal dysfunction through DMT1 degradation; (3) GPX4 is present in the outer membrane of lysosomes and accumulates in the lysosomal membrane; (4) mTOR accumulates in lysosomes; and (5) lysosomal dysfunction is mainly caused by several enzymes of the glycolytic and NAD pathways, including ALDOA, GAPDH, NAMPT, and PGK1, not functioning effectively under glucose starvation.

Under glucose starvation, lysosomes work harder to produce energy,^5^ and selective lysosomal membrane turnover is induced.^10^ Therefore, we postulated that lysosomal function increases under glucose starvation. However, under glucose starvation, there was a decrease in lysosomal-related proteins and an increase in lysosomal damage. Moreover, lysosomal activity (cathepsin D activity), acidification, and degradation decreased. We hypothesized that glucose is essential for lysosomal function.

Autophagy-related proteins (p62 and LC3) are increased,^3^ and up-stream autophagy-related proteins (AMPK and ULK1) are also increased, under glucose starvation.^9^ p62 and LAMP2 staining showed changes in lysosomal morphology, and some p62 and LAMP2 staining merged. These results, including the change in LAMP2 morphology and decreased cathepsin D activity, suggest a decrease in lysosomal function and autophagy. In addition, DALGreen/DAPRed staining showed the accumulation of auto-phagosomes and auto-lysosomes under glucose starvation. Under lysosomal dysfunction conditions, auto-lysosomes accumulate without being degraded. The phenomenon is similar under glucose starvation.^30^

We found that lysosomal function decreased during long-term glucose deficiency, and ferroptosis occurred. Ferroptosis is the most recently discovered mechanism of cell death.^12^ There are some reports^39^ on the occurrence of ferroptosis using elevated mRNA levels. In this study, we used various techniques, including PCR, western blotting, GSSG measurement, and LipiRADICAL Green and FerroOrange staining, to confirm the occurrence of ferroptosis.^35^

Under glucose starvation, both lysosomal dysfunction and ferroptosis occurred. Evidence suggests that lysosomal function is related to ferroptosis.^40^ Nagakannan et al. reported that cathepsin B mediates ferroptosis. In this study, cathepsin B expression decreased under glucose starvation, and the mechanism of glucose starvation-induced ferroptosis was not due to cathepsin B-mediated ferroptosis.^41^ Mai et al. showed that iron accumulation promotes reactive oxygen species production and lysosomal dysfunction.^42^ However, there are no reports on whether lysosomal dysfunction occurs prior to ferroptosis. Accordingly, in this study, we elucidated this phenomenon. Time-course experiments showed that lysosomal function decreased within 12–24 h, after which iron dynamics changed, and ferroptosis occurred at 72 h. We hypothesized that lysosomal dysfunction causes lysosomal iron accumulation, resulting in ferroptosis. One reason for this phenomenon is DMT1 dysfunction, which inhibits lysosomal iron release, because the DMT1 band in western blotting widened and changed.^43^ To confirm this, we used BafA1, which induces DMT1 degradation and ferroptosis. Moreover, DMT1 inhibition induced ferroptosis and lysosomal dysfunction.

Generally, under ferroptotic conditions, GPX4 decreases.^21^^,^^37^ However, in this study, GPX4 increased. Experiments have been conducted involving the inhibition of XCT, which is up-stream of GPX4.^44^ However, our glucose starvation experiments showed that ferroptosis indirectly affected GPX4 expression. Considering the function of GPX4, we hypothesized that GPX4 increases under glucose starvation to protect cells from ferroptosis. GPX4 is present in the nucleus, cytosol, and mitochondria.^22^ We clarified the localization of GPX4 using fractionation experiments. Under glucose starvation, GPX4 accumulates in the outer membrane of lysosomes to protect cells and inhibit ferroptosis. These findings were confirmed by all three GPX4 inhibitors.

Under nutrient deficiency, AMPK is increased to produce energy, whereas mTOR is decreased.^29^ However, in our study, glucose starvation did not decrease mTOR levels. mTOR regulates V-ATPase that produces ATP and lysosomes,^25^ and its hyperactivity causes lysosomal dysfunction.^28^ We found that mTOR was mainly localized in lysosomes and increased under glucose starvation. We consider this increase in mTOR under glucose starvation to be one of the reasons for the decrease in lysosomal function. Rapamycin is an mTOR inhibitor that lowers ferroptosis-related mRNA levels, including PTGS2. Moreover, it decreases LipiRADICAL Green fluorescence. Therefore, rapamycin may positively influence lysosomal-related diseases. Further investigations are required to understand why mTOR increases under glucose starvation.

Around lysosomes, ATP-producing enzymes, including GAPDH and PGK1, are important for lysosomal function.^30^ We hypothesize that lysosomes contain various enzymes, which regulate and maintain lysosomal function. In this study, other enzymes, including ALDOA (an up-stream glycolytic enzyme), were also present in lysosomes. ALDOA, GAPDH, and PGK1 are successive glycolytic enzymes, which may form a complex in the outer membrane of lysosomes, suggesting that the outer membrane of lysosomes is a hub of metabolic signaling. In addition, we extracted lysosomes and analyzed ATP production using F1,6BP (a substrate of ALDOA). Many important enzymes are present in the outer membrane of lysosomes under glucose starvation, owing to the lack of glucose; therefore, these enzymes cannot function effectively, causing lysosomal dysfunction. NAMPT, which regulates the co-enzyme NAD, is also present in lysosomes, and its inhibition by FK866 causes ferroptosis.

To confirm the importance of glucose for lysosomal function, we conducted amino acid starvation experiments. Glutamine is the most abundant free amino acid in animal tissues.^45^^,^^46^ Numerous reports have focused on glutamine starvation.^46^ In this study, we compared glutamine deprivation with glucose starvation. During glutamine deprivation, mature cathepsin B and D expression increased. DALGreen/DAPRed staining showed that lysosomal activity did not decrease (i.e., lysosomal dysfunction did not occur during glutamine deprivation). These results suggest that glucose but not glutamine is important for lysosomal function.

In conclusion, lysosomal function decreased under glucose starvation because mTOR accumulated and glycolytic enzymes around lysosomes could not function effectively. Following lysosomal dysfunction, ferroptosis occurred. Meanwhile, GPX4 accumulated to protect lysosomes from ferroptosis. Lysosomes are associated with various diseases, including lysosomal disease, cancer, and Alzheimer’s disease. There may be a relationship between glucose starvation and lysosomal dysfunction in these diseases. Our findings may be useful for understanding the mechanisms of lysosomal-related diseases.

### Limitations of the study

This study had some limitations. First, many nutrients underlie the lysosomal mechanism of action. In this study, we elucidated how lysosomes maintain their function under glucose starvation. To better understand lysosomal function, a more complex balance of nutrients should be considered in the future. Second, our results should be confirmed in more complex biological systems so that they can be applied to treat lysosomal-related diseases.

## STAR★Methods

### Key resources table

**Table:** 

REAGENT or RESOURCE	SOURCE	IDENTIFIER
**Recombinant DNA**		

TMEM192	Addgene	104434

**Antibodies**		

LC3A/B (D3U4C) XP Rabbit mAb	Cell Signaling Technology	12741
Anti-p62 (SQSTM1) pAb	MBL Life Science	PM045
LAMP-2 Antibody (H4B4)	Santa Cruz Biotechnology	sc-18822
GAPDH (14C10) Rabbit mAb	Cell Signaling Technology	2118
Phospho-ULK1 (Ser555) (D1H4)	Cell Signaling Technology	5869
ULK1 (D8H5) Rabbit mAb	Cell Signaling Technology	8054
Monoclonal anti-HA antibody produced in mouse	Sigma-Aldrich	H3663
Anti-HA-tag mAb-Alexa Fluor 488	MBL Life Science	561-A48
Anti-PGK1 antibody [EPR19057]	Abcam	ab199438
V-ATPase B1/2 Antibody	Santa Cruz Biotechnology	sc-55544
DAPI solution	Dojindo Laboratories	D532
Voltage-dependent anion channel (VDAC)	Yagi et al.^47^	
Monoclonal Anti-β-Actin antibody produced in mouse	Sigma-Aldrich	#A5441
Cathepsin B (D1C7Y) XP Rabbit mAb	Cell Signaling Technology	#31718
Anti-Cathepsin D antibody	Abcam	ab75852
Anti-Galectin 3 antibody	Abcam	ab2785
Cathepsin D Activity Assay Kit	Abcam	ab65302
Phospho-Akt (Ser473) (D9E) XP Rabbit mAb	Cell Signaling Technology	#4060
Phospho-Akt (Thr308) (C31E5E) Rabbit mAb	Cell Signaling Technology	#2965
Akt (pan) (C67E7) Rabbit mAb	Cell Signaling Technology	#4691
Phospho-AMPKα (Thr172)	Cell Signaling Technology	#2535
AMPKα (23A3) Rabbit mAb	Cell Signaling Technology	#2603
Transferrin Receptor Monoclonal Antibody (H68.4)	Invitrogen | Thermo Fisher Scientific	#13-6800
xCT/SLC7A11 (D2M7A) Rabbit mAb	Cell Signaling Technology	#12691
FTH1 Antibody	Cell Signaling Technology	#3998
Anti-DMT1/SLC11A2 rabbit mAb	Cell Signaling Technology	#15083
GPX4 Antibody	Cell Signaling Technology	#52455
Glutathione Peroxidase 4/GPX4 Antibody (Figure 4I)	Santa Cruz Biotechnology	sc-166570
HSP70 Antibody	Cell Signaling Technology	#4872
Phospho-mTOR(Ser2448)	Cell Signaling Technology	#5536
mTOR (7C10) Rabbit mAb	Cell Signaling Technology	#2983
Aldolase A1 Rabbit mAb	Cell Signaling Technology	#8060
Nampt	Our laboratory	
Anti-PGK1 antibody	Abcam	ab199438

**Chemical, enzymes, and other reagents**		

Daporinad (FK866)	Selleckchem	S2799
Bafilomycin A1 (NSC381866)	Bioviotica Naturstoffe GmbH	NSC381866
DQ Green BSA	Thermo Fisher Scientific	D12050
DAPRed	Dojindo Laboratories	D675
DALGreen	Dojindo Laboratories	D676
Lipi RADCIALGreen	Dojindo Laboratories	FDV0042
FerroOrange	Dojindo Laboratories	F374
Deferoxamine mesylate salt	Sigma-Aldrich	205-314-3
(1S,3R)-RSL3	Cayman Chemical	19288
GSSG/GSH Quantification Kit	Dojindo Laboratories	342-09011
ML-162	Cayman Chemical	20455
M-L210	Cayman Chemical	23282
LysoTracker Red	Invitrogen | Thermo Fisher Scientific	7528
LysoPrime Green	Invitrogen | Thermo Fisher Scientific	L261
Cathepsin D Activity Assay Kit	Abcam	ab65302
Dextran, Tetramethylrhodamine	Invitrogen | Thermo Fisher Scientific	D1868
Dextran, Oregon Green	Invitrogen | Thermo Fisher Scientific	D7170
Adenosine diphosphate (ADP)	Sigma-Aldrich	A2754
Nicotinamide adenine dinucleotide (NAD)	Sigma-Aldrich	N0632
Sodium iodoacetate	Sigma-Aldrich	I2512
D-Fructose-1,6-diphosphate trisodium salt octahydrate	Selleckchem	S6290
Ferrostatin-1	cayman Chemical	17729
DMT1 blocker 2	MedChemExpress	HY-126302
Dulbecco’s modified Eagle’s medium (DMEM; No Glucose)	Nacalai	09891-25
Fetal bovine serum (FBS)	Sigma-Aldrich	F7524
Glucose	FUJIFILM Wako Pure Chemical Corporation	079-05511
penicillin-streptomycin	Nacalai	09367-34
Pyruvate solution	Nacalai	06977-34
Lipofectamine	Invitrogen | Thermo Fisher Scientific	15338-100
Sucrose	FUJIFILM Wako Pure Chemical Corporation	195-07925
Trizma base	Sigma-Aldrich	T6066
Sodium Chloride	WAKO	191-01665
4%paraformaldehyde	nacalai	09154-85
NP-40	FUJIFILM Wako Pure Chemical Corporation	145-09701
Triton-X100	Kishida chemical CO.LTD.	020-81155
Methanol	FUJIFILM Wako Pure Chemical Corporation	137-01823
Hanks' balanced salt solution (HBSS)	FUJIFILM Wako Pure Chemical Corporation	084-08965
Rneasy miki kit	Quiagen	74104
PrimeScript RT Reagent Kit	Takara Bio Inc.	Cat# RR036A
Protease inhibitor	FUJIFILM Wako Pure Chemical Corporation	161-19511
phosphatase inhibitor	sigma-Aldrich	4906837001
Blocking one	nacalai	03953-95
Can get signal	toyobo	F0991K
SPECTROstar Nano software Version 2.10	BMG LABTECH	https://www.bmglabtech.com/jp/microplate-reader-software/
ImageQuant LAS 4000 Version 1.2	GE Healthcare	https://imagequant-las-4000.software.informer.com/1.2/
StepOnePlus Real-Time PCR Systems	Thermo Fisher Scientific	https://www.thermofisher.com/jp/ja/home/technical-resources/software-downloads/StepOne-and-StepOnePlus-Real-Time-PCR-System.html
ImageQuant™ LAS 4000	GE healthcare	GE Heathcare Systems

### Resource availability

#### Lead contact

Takeshi Uchiumi, M.D., Ph.D.

Department of Clinical Chemistry and Laboratory Medicine, Graduate School of Medical Sciences, Kyushu University, Higashi-ku, Fukuoka 812–8582, Japan Tel: +81-92-642-5750 Fax: +81-92-642-5772.

#### Materials availability

All unique/stable reagents generated in this study are available from the lead contact with a completed Materials Transfer Agreement.

#### Data and code availability

• All data reported in this paper will be shared by the lead contact on request.
• This paper does not report original code.
• Any additional information required to reanalyze the data reported in this paper is available from the lead contact on request.
• This study did not generate any unique datasets or code.

### Experimental model and study paricipant details

#### Cell culture

U87MG (U87) cells were obtained from the American Type Culture Collection (Manassas, VA, USA) (certified by BEX [Japan]). MEF and SH-SY5Y cells were obtained from our laboratory.^30^ U87 transfected TMEM192 were constructed as previously reported^30^: the cells were cultured as previously reported in Dulbecco’s modified Eagle’s medium (DMEM, Nacalai Tesque, Japan) containing 10% fetal bovine serum (Sigma-Aldrich, USA) and 1% penicillin–streptomycin (Nacalai Tesque).^35^ Cells were cultured in a humidified incubator with 95% air and 5% CO_2_ at 37°C.

### Method details

#### Reagents

The reagents used in the study are listed in the key resources table.

#### Quantitative real-time PCR

Real-time PCR was performed as previously described.^35^ The total RNA was extracted from cell lines using the RNeasy Mini Kit (Qiagen, Germany). According to the manufacturer’s instructions, the RNA samples were reverse-transcribed using the PrimeScript RT Reagent Kit (Takara Bio, Japan). mRNA expression was detected using qPCR with a thermal cycler (StepOnePlus; Applied Biosystems). Ribosomal 18S rRNA was evaluated as an internal control. The primer sequences are listed in Table S1.

#### Immunoblotting analysis

Immunoblotting was performed as described previously.^35^ The cells were homogenized in lysis buffer (20 mM Tris-HCL, 2 mM EDTA, 150 mM NaCl, and 1% NP40; pH 7.5), which contained protease (Fujifilm Wako, 161–26021, Japan) and phosphatase inhibitors (Sigma-Aldrich, 4906837001). After sonication, the cell lysates were centrifuged at 20,000 ×*g* for 5 min. The supernatants were collected as samples. Equal amounts of protein (5 μg) were separated using SDS-PAGE and transferred onto Immobilon-P transfer membranes (EMD Millipore Corporation, Germany). The membranes were blocked using Blocking One (Nacalai Tesque) and then probed overnight with primary antibodies. The membranes were incubated with secondary antibodies. Proteins were detected by enhanced chemiluminescence (GE Healthcare, UK). Chemiluminescence was recorded and quantified using a chilled-charge-coupled device camera (LAS1000plus). The antibodies used in this study are listed in the key resources table. The membranes were cut prior to antibody hybridization.

#### Cell number counts

Cells were seeded (1 × 10^5^ cells/well in a 6-well dish) in triplicate and cultured in DMEM (containing glucose) with different drug and glucose concentrations for 72 h. The cells were trypsinized and counted using a Coulter counter (Beckman Coulter, USA).

#### DQ-BSA

DQ Green BSA (Thermo Fisher Scientific) was used to assess the lysosomal protease activity. A total of 1 × 10^4^ cells/well were cultured overnight in 96-well plates. DQ Green BSA (50 μg/mL) was added and incubated for 1 h at 37°C, the culture medium was then replaced with PBS, and the fluorescence at 495 nm excitation and 525 nm emission was measured using ARVO (PerkinElmer).

#### Cathepsin D activity assay

We calculated the activity of cathepsin D using a cathepsin D assay kit (ab65302) and the following protocol. Cells (1 × 10^6^) were harvested and resuspended in 200 μL chilled CD lysis buffer. The cells were incubated for 10 min and then centrifuged for 5 min at 4°C and 20,000 ×*g*. Samples in each well (20 μL) were supplemented with 50 μL CD reaction butter and 2 μL substrate and incubated at 37°C for 1 h. Subsequently, a fluorometric assay was conducted using EnSight (PerkinElmer). (Excitation/Emission = 328/460 nm).

#### GSSG assay

Cells (3 × 10^6^) were collected by centrifugation at 200 ×*g* for 10 min at 4°C, washed with 300 μL of PBS, and centrifuged again at 200 ×*g* for 10 min at 4°C. HCl (80 μL, 10 mmol/L) was added to the cells and then they were lysed by freezing and thawing twice, followed by addition of 20 μL of 5% 5-Sulfosalycylic Acid Dihydrate and then centrifuged at 8,000 ×*g* for 10 min. GSSG sample or GSH sample (20 μL) was added to each well. Subsequently, 30 μL of buffer solution was added and the solution incubated at 37°C for 1 h, and then 30 μL of substrate working solution and 30 μL of enzyme/coenzyme working solution were added to each well and incubated at 37°C for 10 min. The absorbance was read at 405 nm using ARVO (PerkinElmer). Finally, the concentration of GSSG was determined using a GSSG calibration curve and the concentration of total glutathione was determined using a GSH calibration curve.

#### Immunoprecipitation

Cells were harvested using trypsin, homogenized in homogenization buffer (250 mM sucrose, 20 mM HEPES-KOH, pH 7.4, 1 mM EDTA, and protease inhibitor cocktail), and centrifuged at 800 ×*g* for 10 min to remove the nuclei. The supernatant was centrifuged at 20,000 ×*g* for 30 min, and the pellet (membrane fraction) was resuspended in 280 mL (mixing at a ratio of 8:1 [Buffer A (1 mM HEPES-KOH, pH 7.2, 15 mM KCl, 1.5 mM MgAc, 1 mM DTT, protease inhibitor cocktail) and B (220 mM HEPES-KOH, pH 7.2, 375 mM KCl, 22.5 mM MgAc, 1 mM DTT, protease inhibitor cocktail]). Adjustment was then carried out so that protein concentration was constant, following by incubation at 4°C. After 3 h of rotation, the beads were washed three times with Buffer AB and eluted with SDS-PAGE sample buffer.^30^

#### Lysosomal fraction

Cells were harvested using trypsin, homogenized in homogenization buffer (250 mM sucrose, 20 mM HEPES-KOH, pH 7.4, 1 mM EDTA, and protease inhibitor cocktail), and centrifuged at 800 ×*g* for 10 min to remove the nuclei. The supernatant was centrifuged at 20,000 ×*g* for 30 min, and the pellet (membrane fraction) was resuspended in 19% (v/v) OptiPrep solution (Axis-Shield Density Gradient Media-Alere Technologies AS) as described previously.^30^^,^^38^ The sample was loaded on a discontinuous density gradient with 27%, 22.5%, 19%, 16%, 12%, and 8% OptiPrep (2 mL each) in a 12-mL centrifugation tube and subjected to ultracentrifugation at 13,000 ×*g* for 4 h at 4°C in a swinging bucket rotor (SW 40.1; Beckman Coulter). Subcellular fractions were collected from the top of the tube, washed, concentrated in homogenization buffer, and centrifuged at 20,000 ×*g* for 30 min. The protein contents of the individual fractions were determined by western blotting.

#### GAPDH and GpX4 binding to lysosomes

To analyze GpX4 and GAPDH binding to lysosomal membranes, purified lysosomal fractions (Fraction No. 2) were divided into five parts and resuspended in homogenization buffer (250 mM sucrose, 20 mM HEPES-KOH, pH 7.4, 1 mM EDTA) containing 0, 0.1, 0.2, 0.4, or 0.8 M NaCl. The mixtures were incubated for 30 min on ice and subjected to ultracentrifugation at 160,000 ×*g* for 1 h at 4°C in a fixed angle rotor (TLA120.2; Beckman Coulter). The pellet (lysosomal membrane) was resuspended in 50 μL of SDS-PAGE sample buffer and resolved by SDS-PAGE. The supernatant (protein from the lysosomal membrane) was added to 20% trichloroacetic acid (TCA) to extract protein for 30 min and centrifuged at 20,000 ×*g* for 10 min at 4°C. The pellet (extract protein) was washed with cold acetone and centrifuged at 20,000 ×*g* for 5 min at 4°C. The TCA/acetone precipitate was also suspended in 50 μL of SDS-PAGE sample buffer and resolved by SDS-PAGE.^30^

#### Lysosomal content assay

Cells were stained with Dextran, Oregon Green 488, 10,000 MW, Anionic (quenched under acidic environment) and Dextran, Tetramethylrhodamine, 10,000 MW, Anionic, Lysine Fixable (pH-independent) (Thermo Fisher) to measure lysosomal acidification. After addition of the two dextran molecules, cells were incubated for 8 h at 37°C in a glass bottom dish (Iwaki) and incubated overnight after medium change.^30^ The medium of the labeled cells was replaced with HBSS, and the cells were observed under a fluorescence microscope. The intensity of green and red fluorescence was measured and calculated (BZ-800, Keyence), enabling changes in lysosomal pH to be monitored.

#### Fluorescence probes

DALGreen and DAPRed (Dojindo) were used to stain autophagosomes with enhanced fluorescence in hydrophobic environments. DALGreen fluorescence is enhanced at an acidic pH and is suitable for monitoring the autophagy degradation stage (autolysosomes). In contrast, DAPRed has a pH-independent fluorescence profile and remains fluorescent at an almost constant intensity throughout autophagy (autophagosome). Cells seeded in glass bottom dishes were stained with DALGreen and DAPRed for 30 min at 37°C. The medium was replaced with HBSS and the intensities of the green and red signals were measured and calculated (BZ-X800, Keyence).

#### Immunostaining

Cells seeded in glass bottom dishes (when observed after 24 h, 2 × 10^4^ cells were seeded; after 72 h, 1 × 10^4^ cells were seeded) were stained with LipiRADICAL Green (1 μM) for 10 min, Ferro Orange (1 μM) for 30 min, DQ-BSA (50 μg/mL) for 60 min, LysoTracker red (1 μM) for 60 min, and LysoPrime Green for 30 min at 37°C. The medium was replaced with a low-glucose medium or HBSS (Fujifilm Wako, 084–08965) before staining. Signal intensities were measured and calculated (calculated average luminance [total luminance/cell number]) in each image (*N* = 10) (BZ-X800; KEYENCE).

#### Immunohistochemistry

U87 cells were incubated in each condition for 72 h in 5% CO_2_ at 37°C. Immunofluorescence was carried out according to established techniques.^30^ U87 cells were fixed with 4% paraformaldehyde phosphate buffer solution (Nacalai Tesque) for 10 min and permeabilized in 0.2% Triton X-100/PBS for 5 min. Samples were blocked with Blocking One (Nacalai Tesque), and the primary antibody was diluted with Can Get Signal® (Toyobo).

#### Aldolase, GAPDH, and PGK1-dependent ATP production

Cells were harvested using trypsin, homogenized in homogenization buffer (250 mM sucrose, 20 mM HEPES-KOH, pH 7.4, 1 mM EDTA, and protease inhibitor cocktail), and centrifuged at 800 ×*g* for 10 min to remove the nuclei. The supernatant was centrifuged at 10,000 ×*g* for 3 min, and the pellet containing lysosomes was resuspended in 150 μL of reaction buffer (20 mM HEPES-KOH, pH 7.2, 200 mM sucrose, 50 mM KCL, 0.1 mM NAD, 0.05 mM ADP, 0.1 mM KH_2_PO_4_). To measure ATP production, 1 mM F1,6-BP and 10 μM iodoacetate, which is a GAPDH inhibitor, were added. The reaction was allowed to occur at 37°C for 5 min. Subsequently, 100 μL of the reaction mixture and 100 μL of the CellTiter-Glo reagent were mixed in a 96-well plate. After 10 min, luminescence was recorded (CellTiter-Glo Luminescent Cell Viability Assay (Promega)).^48^

### Quantification and statistical analysis

Data are presented as mean ± standard deviation. Significant differences between the groups were examined using a one-way analysis of variance or Student’s t test with GraphPad Prism version 9 (GraphPad Prism Software Inc.). All experiments were repeated at least three times.
