# Supplementary material for: Glucose starvation causes ferroptosis-mediated lysosomal dysfunction
Source: iScience. 2024 Apr 12;27(5):109735. doi: 10.1016/j.isci.2024.109735 (PMC11067335; doi:10.1016/j.isci.2024.109735)
Supplement: Document S1. Figures S1–S5 and Table S1 [file mmc1.pdf]

iScience, Volume *27*

Supplemental information

Glucose starvation causes ferroptosis-mediated lysosomal dysfunction

Kenji Miki, Mikako Yagi, Dongchon Kang, Yuya Kunisaki, Koji Yoshimoto, and Takeshi Uchiumi

Supplemental Information for:

Glucose Starvation Causes Ferroptosis-Mediated Lysosomal Dysfunction

Kenji Miki[1,2,6], Mikako Yagi[1,3,6], Dongchon Kang[1,4,5], Yuya Kunisaki[1], Koji Yoshimoto[2], and Takeshi Uchiumi[1,3,7*]

[1]Department of Clinical Chemistry and Laboratory Medicine, Graduate School of Medical Sciences, Kyushu University, Higashi-ku, Fukuoka 812-8582, Japan

[2]Department of Neurosurgery, Graduate School of Medical Sciences, Kyushu University, Higashi-ku, Fukuoka 812-8582, Japan

[3]Department of Health Sciences, Graduate School of Medical Sciences, Kyushu University, Higashi-ku, Fukuoka 812-8582, Japan

[4]Kashiigaoka rehabilitation hospital, Fukuoka 813-0002, Japan

[5]Department of Medical Laboratory Science, Faculty of Health Sciences, Junshin Gakuen University, Fukuoka 815-8510, Japan

[6]These authors contributed equally

***Corresponding author and [7]Lead contact:** Takeshi Uchiumi, M.D., PhD.

Department of Clinical Chemistry and Laboratory Medicine, Graduate School of Medical Sciences, Kyushu University, Higashi-ku, Fukuoka 812-8582, Japan

Tel: +81-92-642-5750

Fax: +81-92-642-5772

E-mail: uchiumi.takeshi.008@m.kyushu-u.ac.jp

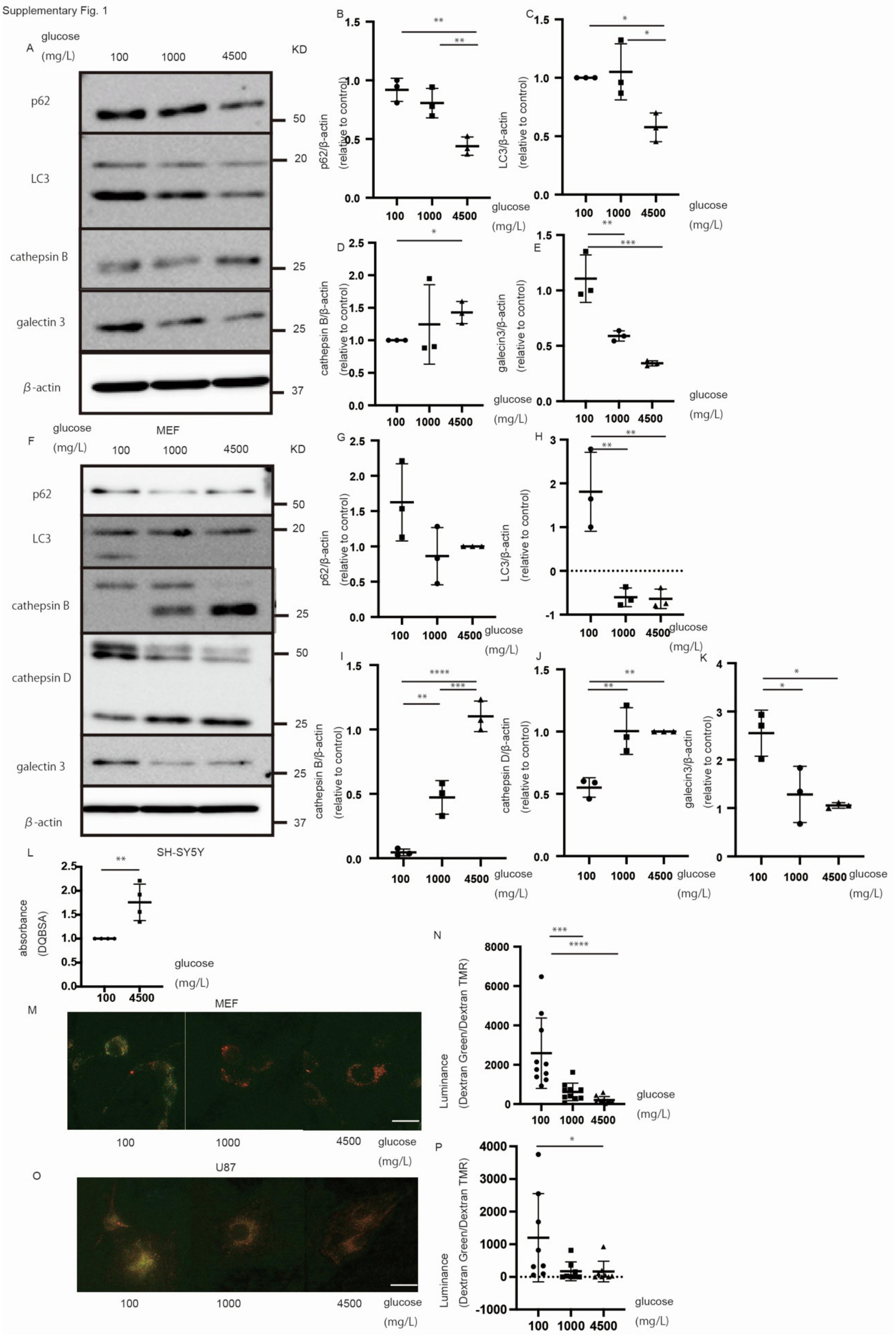
Supplementary Fig. 1
A
glucose (mg/L)
100 1000 4500
KD
p62
LC3
cathepsin B
galectin 3
β-actin
50
20
25
25
37
B
p62/β-actin (relative to control)
C
LC3/β-actin (relative to control)
D
cathepsin B/β-actin (relative to control)
E
galecin3/β-actin (relative to control)
F
MEF
cathepsin D
G
H
I
J
cathepsin D/β-actin (relative to control)
K
L
SH-SY5Y
absorbance (DQBSA)
M
N
Luminance (Dextran Green/Dextran TMR)
O
U87
P

Figure S1. **Expression and activity of lysosomal proteins decreased under glucose starvation.**

A Expression of autophagy- and lysosomal-related proteins (p62, LC3, cathepsin B, and LGALS3) in MEF cells under each glucose concentration for 72 h.

B–E Quantification of (B) p62, (C) LC3, (D) cathepsin B, and (E) LGALS3.

F Expression of autophagy- and lysosomal-related proteins (p62, LC3, cathepsin B and D, and LGALS3) in SH-SY5Y cells under each glucose concentration for 72 h.

G–K Quantification of (G) p62, (H) LC3, (I) cathepsin B, (J) cathepsin D, and (K) LGALS3.

L Lysosomal activity of DQ-BSA decreases under glucose starvation under each glucose concentration for 72 h in U87 cells.

M Lysosomal acidification is impaired under glucose starvation. Oregon Green 488–dextran is quenched under acidic pH; thus, an increased green:red ratio

denotes impaired lysosomal acidification in MEF cells under each glucose concentration for 72 h in U87 cells.

N Quantification of Oregon Green 488–dextran/TMR–dextran.

O Lysosomal acidification is impaired under glucose starvation in U87 cells under each glucose concentration for 72 h.

P Quantification of Oregon Green 488–dextran/TMR–dextran. Scale bars: 20 μm. Values are presented as mean ± SD. Student's *t*-test (D, L) or one-way ANOVA and Tukey's multiple comparison test (B, C, E, G–K, M–P) was performed to assess 100 *vs*. 1,000 *vs*. 4,500 mg/L of glucose. $^{*}P < 0.05$; $^{**}P < 0.01$; $^{***}P < 0.001$; $^{****}P < 0.001$.

Supplementary Fig. 2

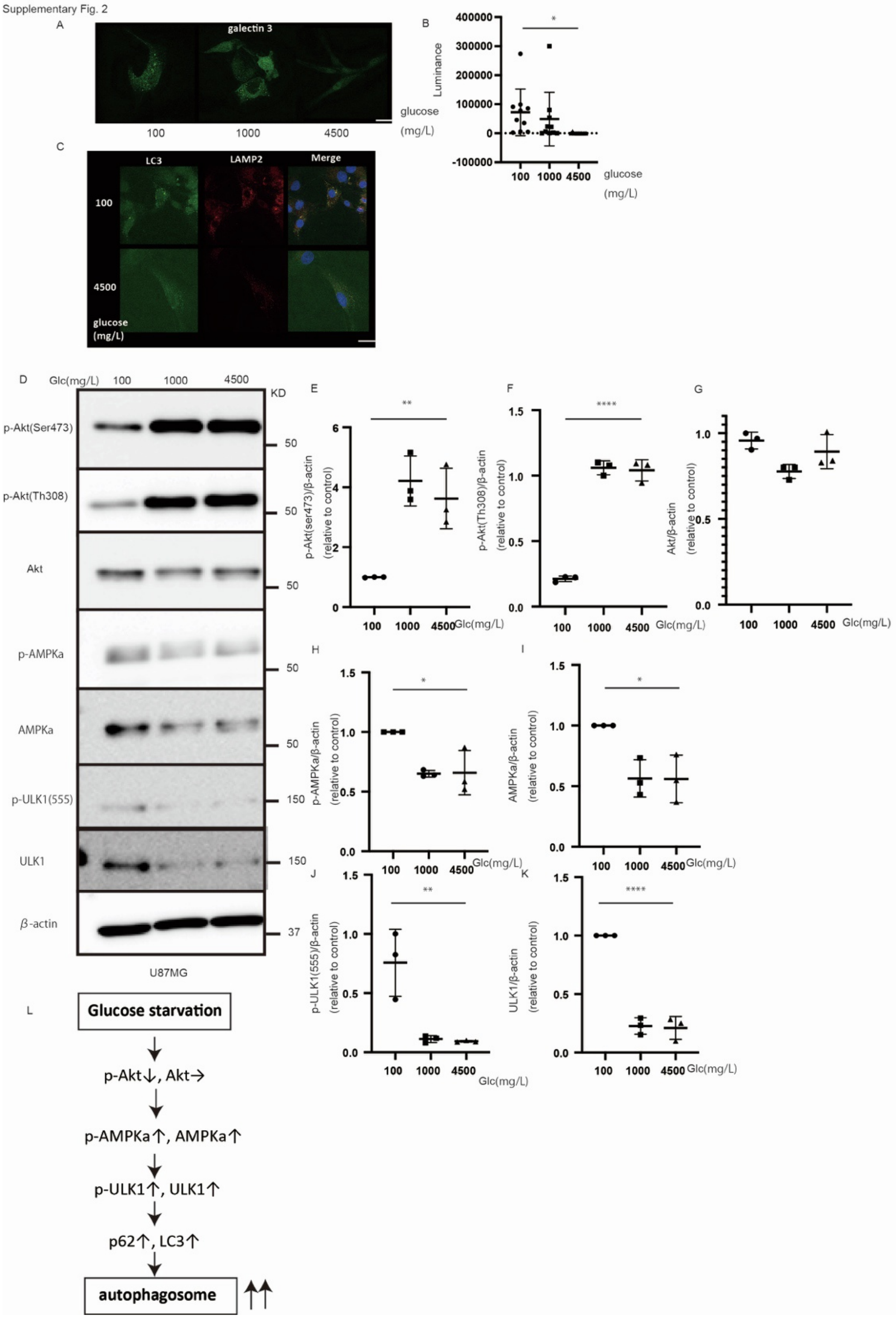

53

Figure S2. **Lysosomal morphology changes under glucose starvation and processes up-stream of autophagy initiation under glucose starvation in U87 cells.**

A Morphology of LGALS3 under glucose starvation for 72 h.

B Quantification of LGALS3.

C Morphology of LC3 under glucose starvation. Scale bars: 20 μm.

D Expression of AKT-related, AMPK-α, and ULK1 proteins in U87 cells under each glucose concentration for 72 h.

E–K Quantification of (E) p-AKT (Ser473), (F) p-AKT (Th308), (G) AKT, (H) p-AMPK-α (Thr172), (I) AMPK-α, (J) p-ULK1, and (K) ULK1.

L Schematic of up-stream autophagy initiation. Values are presented as mean ± SD. One-way ANOVA and Tukey’s multiple comparison test were performed to assess 100 *vs*. 1,000 *vs*. 4,500 mg/L of glucose. $^{*}P < 0.05$; $^{**}P < 0.01$; $^{****}P < 0.0001$.

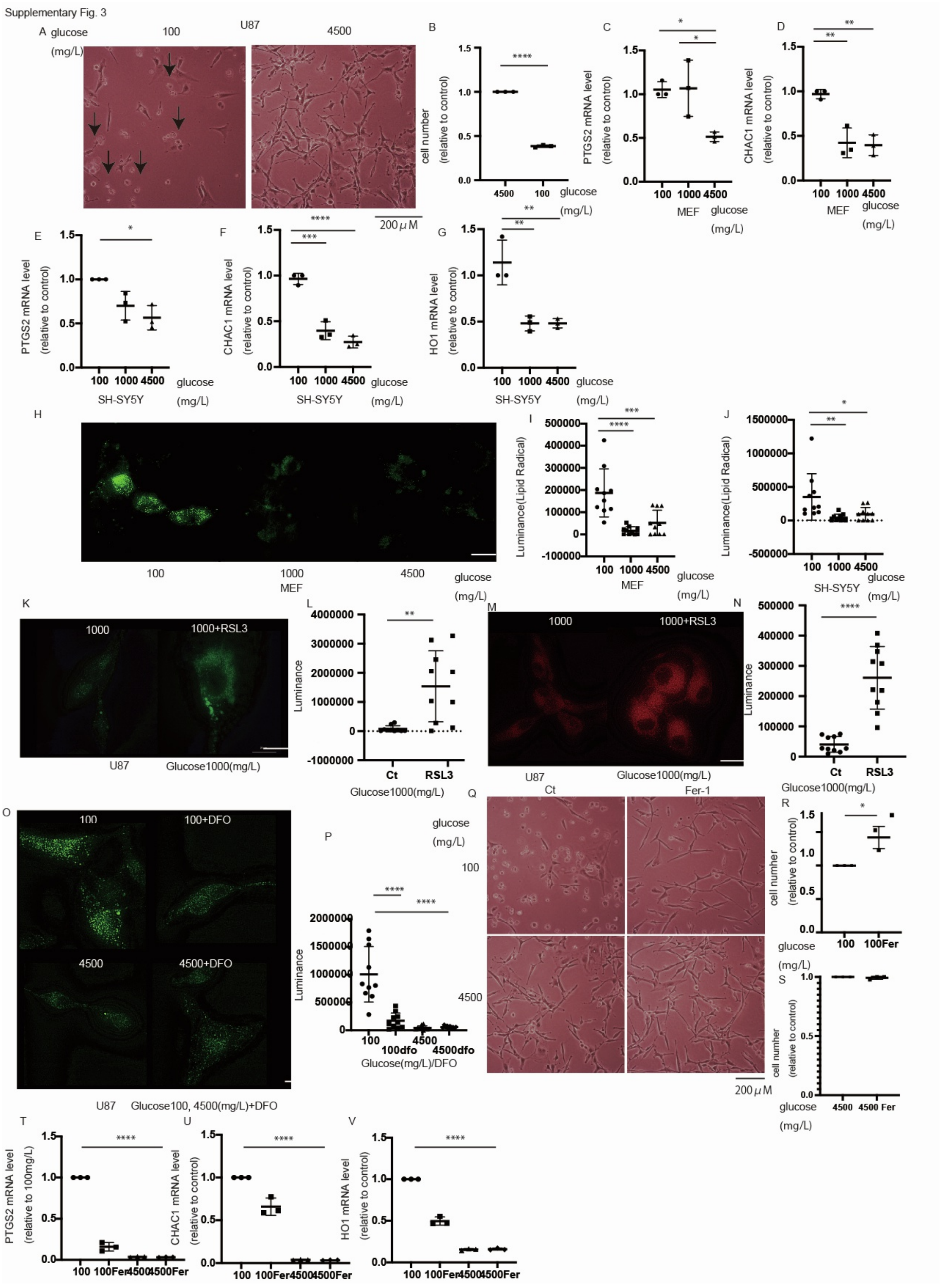

Figure S3. **Ferroptosis is induced as a result of glucose starvation or by RSL3 via**

lipid radicals and FerroOrange.

A,B Under glucose-starved conditions for 72 h, U87 cells became weak and exhibited detachment (arrow) (**A**). Quantification of cell number (**B**).

Under glucose starvation for 72 h, ferroptosis markers increased in both MEF and SH-SY5Y cells.

C,D Quantification of (C) PTGS2 and (D) CHAC1 in MEF cells.

E-G Quantification of (E) PTGS2, (F) CHAC1, and (G) HO-1 in SH-SY5Y cells.

H LipiRADICAL Green fluorescence was higher under glucose starvation in MEF cells.

I Quantification of LipiRADICAL Green fluorescence.

J LipiRADICAL Green fluorescence was higher under glucose starvation in SH-SY5Y cells. Scale bars: 20 μm. Values are presented as mean ± SD. Student’s *t*-test (B) or one-way ANOVA and Tukey’s multiple comparison test (C, E-G, I, J) were performed to assess 100 *vs*. 1,000 *vs*. 4,500 mg/L of glucose. $^{*}P < 0.05$; $^{**}P < 0.01$; $^{***}P < 0.001$; $^{****}P < 0.0001$.

K LipiRADICAL Green fluorescence was higher after RSL3 injection for 72 h in U87 cells.

L Quantification of LipiRADICAL Green fluorescence.

M FerroOrange fluorescence was determined using RSL3 in U87 cells.

N Quantification of FerroOrange fluorescence.

O LipiRADICAL Green fluorescence was higher under glucose starvation for 72 h; however, DFO (5 μM; 24 h) reduced LipiRADICAL Green fluorescence in U87 cells.

P Quantification of LipiRADICAL Green fluorescence. Scale bars: 20 μm.

Q Glucose-starved and normal conditions with or without Fer1 (40μM).

R,S Quantification of cell number calculated by trypan blue (relative to control (10μM/control)).

T,U,V Quantification of (T) PTGS2 and (U) CHAC1 (V) HO-1 in U87 cells with or without Fer-1 (2μM) under each glucose concentration.

Values are presented as mean ± SD. Student's *t*-test (L, N) or one-way ANOVA and Tukey's multiple comparison test (P) was performed to assess

control *vs*. RSL3, 100 *vs*. 4,500 mg/L of glucose with or without DFO or Fer1. $^{**}P < 0.01$; $^{****}P < 0.0001$.

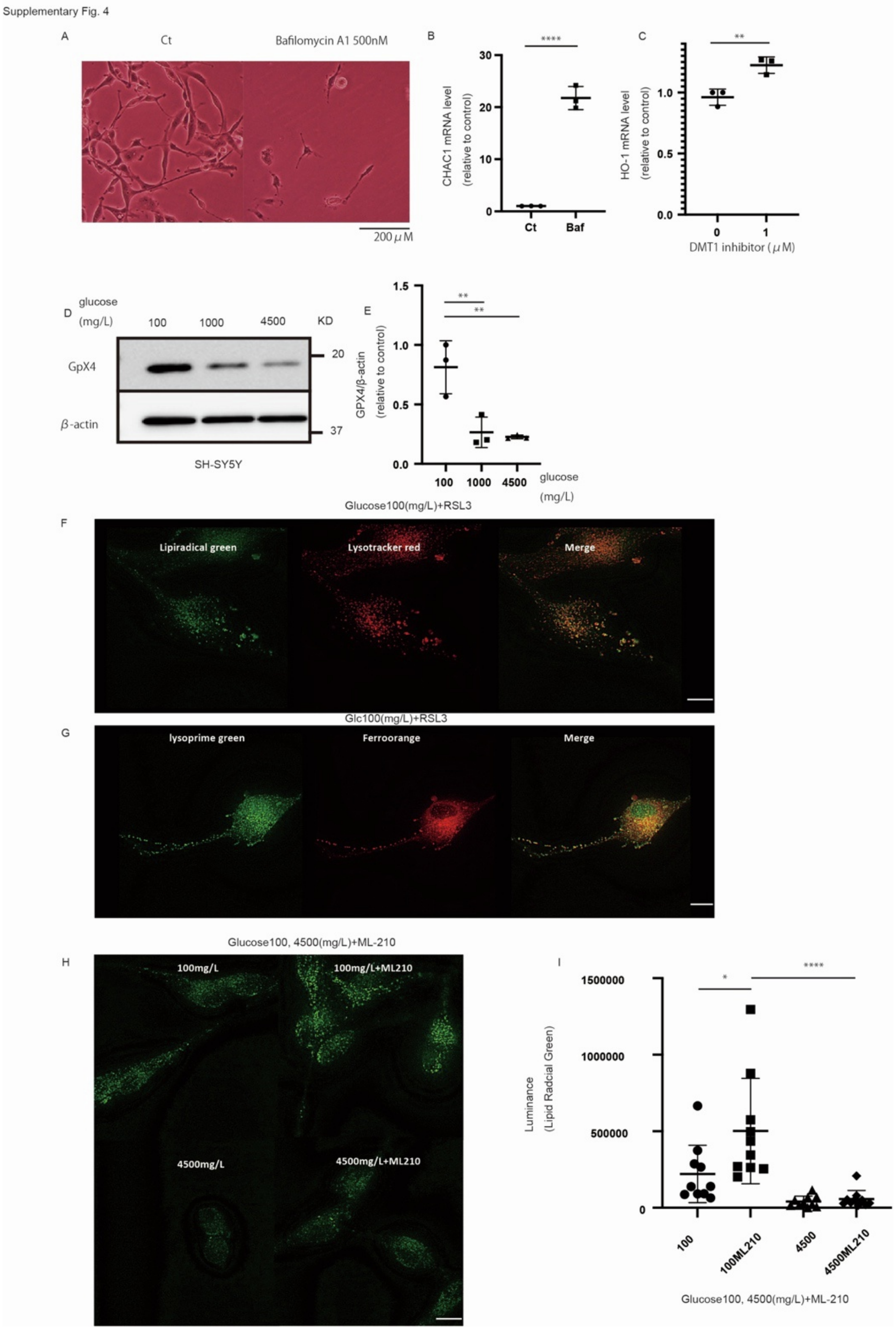

Figure S4. **BafA1 induces ferroptosis, and LipiRADICAL Green accumulates in lysosomes.**

A Cells died when treated with BafA1 (500 nM; 48 h) in U87 cells.

B Quantification of CHAC1 using BafA1.

C Quantification of HO-1 using DMT1 blocker (1 μM; 72 h). Values are presented as mean ± SD. Student’s *t*-test (B, C) was performed to assess control *vs*. BafA1, or control *vs*. DMT1 inhibitor. $^{****}P < 0.0001$.

D Expression of GPX4 under each glucose concentration for 72 h in SH-SY5Y cells.

E Quantification of GPX4. Values are presented as mean ± SD. One-way ANOVA and Tukey’s multiple comparison test (E) were performed to assess 100 *vs*. 1,000 *vs*. 4,500 mg/L of glucose. $^{**}P < 0.01$.

F LipiRADICAL Green and LysoTracker Red co-localisation in U87 cells.

G LysoPrime Green and FerroOrange co-localisation.

H Increase in LipiRADICAL Green fluorescence using ML210 (5 nM; 48 h), especially under glucose starvation.

I Quantification of LipiRADICAL Green fluorescence. Scale bars: 20 μm. Values are presented as mean ± SD. One-way ANOVA and Tukey's multiple comparison test (I) were performed to assess 100 *vs*. 4,500 mg/L of glucose with or without ML210. $^{*}P < 0.05$; $^{****}P < 0.001$; $^{****}P < 0.0001$.

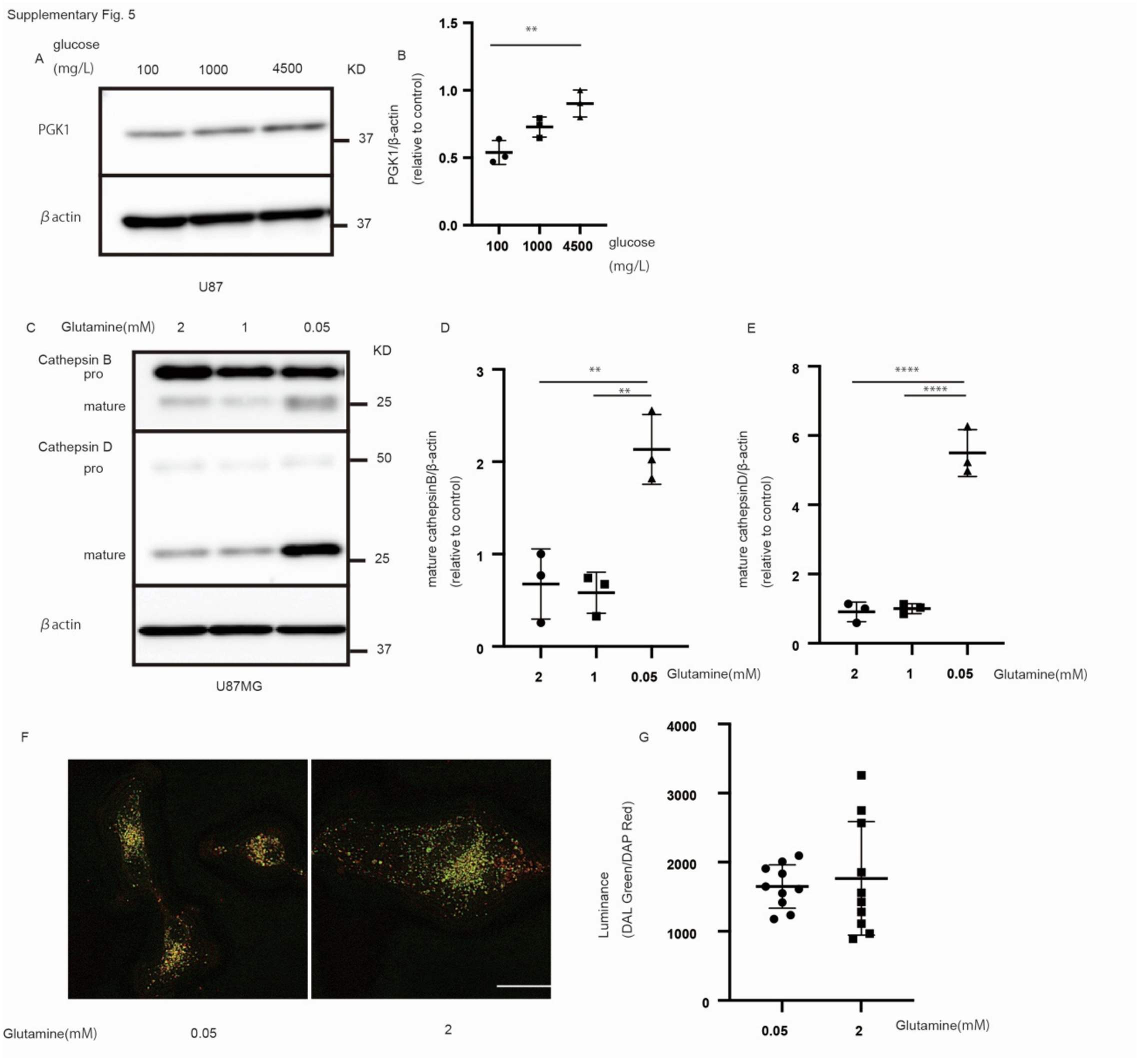

Figure S5. **PGK1 decreases under glucose starvation, and lysosomal function does not decrease under glutamine starvation.**

A Expression of PGK1 under each glucose concentration for 72 h.

B Quantification of PGK1. Values are presented as mean ± SD. One-way ANOVA and Tukey's multiple comparison test (B) were performed to assess 100 *vs*. 1,000 *vs*. 4,500 mg/L of glucose. $^{**}P < 0.01$.

C Expression of lysosomal proteins, including cathepsin B and D, under each glutamine concentration for 72 h.

D, E Quantification of (D) cathepsin B and (E) cathepsin D.

F DALGreen fluorescence was higher and the cells created sufficiently acidic conditions.

G Quantification of DALGreen/DAPRed. Scale bars: 20 μm. Values are presented as mean ± SD. Student's *t*-test (G) or one-way ANOVA and Tukey's multiple comparison test (D, E) was performed to assess 0.05 *vs*. 1 *vs*. 2 mM of glutamine. $^{**}P < 0.01$; $^{****}P < 0.0001$.

Table S1. Primer sequences used in the present study

**Primer**		
**Target**	**Forward (5′→3′)**	**Reverse (3′→5′)**
18S	AAACGGCTACCACATCCAAG	CCTCCAATGGATCCTCGTTA
PTGS2	TGAGCATCTACGGTTTGCTG	TGCTTGTCTGGAACAACTGC
CHAC1	AGATCATGAGGGCTGCACTT	CCAGACGCAGCAAGTATTCA
HO-1	GGC CTC CCTGTACCACATCT	AGACAGGTCACCCAGGTAGC
